# *Leptosphaeria maculans* Alters Glucosinolate Profiles in Blackleg Disease–Resistant and -Susceptible Cabbage Lines

**DOI:** 10.3389/fpls.2017.01769

**Published:** 2017-10-12

**Authors:** Arif Hasan Khan Robin, Go-Eun Yi, Rawnak Laila, Mohammad Rashed Hossain, Jong-In Park, Hye R. Kim, Ill-Sup Nou

**Affiliations:** ^1^Department of Horticulture, Sunchon National University, Suncheon, South Korea; ^2^Department of Genetics and Plant Breeding, Bangladesh Agricultural University, Mymensingh, Bangladesh; ^3^Plant Systems Engineering Research Center, Korea Research Institute of Bioscience and Biotechnology, Daejeon, South Korea

**Keywords:** blackleg disease, *Leptosphaeria maculans*, glucosinolates, resistance, cabbage, expression analysis

## Abstract

Blackleg, a fungal disease caused by *Leptosphaeria maculans*, is one of the most devastating diseases of *Brassica* crops worldwide. Despite notable progress elucidating the roles of glucosinolates in pathogen defense, the complex interaction between *B. oleracea* (cabbage) and *L. maculans* infection that leads to the selective induction of genes involved in glucosinolate production and subsequent modulation of glucosinolate profiles remains to be fully understood. The current study was designed to identify glucosinolate-biosynthesis genes induced by *L. maculans* and any associated alterations in glucosinolate profiles to explore their roles in blackleg resistance in 3-month-old cabbage plants. The defense responses of four cabbage lines, two resistant and two susceptible, were investigated using two *L. maculans* isolates, 03–02 s and 00–100 s. A simultaneous increase in the aliphatic glucosinolates glucoiberverin (GIV) and glucoerucin (GER) and the indolic glucosinolates glucobrassicin (GBS) and neoglucobrassicin (NGBS) was associated with complete resistance. An increase in either aliphatic (GIV) or indolic (GBS and MGBS) glucosinolates was associated with moderate resistance. Indolic glucobrassicin (GBS) and neoglucobrassicin (NGBS) were increased in both resistant and susceptible interactions. Pearson correlation showed positive association between GER content with *GSL-OH* (*Bol033373*) expression. Expressions of *MYB34* (*Bol007760*)*, ST5a* (*Bol026200*), and *CYP81F2* (*Bol026044*) were positively correlated with the contents of both GBS and MGBS. Our results confirm that *L. maculans* infection induces glucosinolate-biosynthesis genes in cabbage, with concomitant changes in individual glucosinolate contents. In resistant lines, both aliphatic and indolic glucosinolates are associated with resistance, with aliphatic GIV and GER and indolic MGBS glucosinolates particularly important. The association between the genes, the corresponding glucosinolates, and plant resistance broaden our molecular understanding of glucosinolate mediated defense against *L. maculans* in cabbage.

## Introduction

Blackleg is one of the most devastating diseases of *Brassica* crop species, causing an estimated $900 million of crop losses throughout the world every year (Howlett, 2004; Fitt et al., 2006, 2008). Blackleg is caused by the hemi-biotrophic fungal pathogen *Leptosphaeria maculans*. Severe blackleg infection can result in complete loss of *Brassica napus* canola or oilseed rape crops (Li et al., 2003; Rouxel et al., 2003; Sprague et al., 2006). Blackleg is similarly devastating for vegetable varieties of *B. oleracea*, including cabbage (Humpherson-Jones, 1985; Rico et al., 2001; Dilmaghani et al., 2010, 2013; Piliponyte-Dzikiene et al., 2015); in fact, the first report of a blackleg epidemic in cabbage (in Wisconsin, USA) is almost a century old (Henderson, 1918). Despite the availability of various chemical control measures (Del Rio and Ruud, 2013; Fraser et al., 2016; Koh et al., 2016), development of effective resistance through breeding remains the most widely accepted means of protecting *Brassica* germplasm.

In addition to race-specific resistance conferred by *R* genes, secondary metabolites produced by the plants also offer more general resistance against pathogens and insects (Wink, 1988; Giamoustaris and Mithen, 1997; Tierens et al., 2001; Brader et al., 2006; Lattanzio et al., 2006). Glucosinolates are Sulfur- and nitrogen-containing secondary metabolites that are the precursors of isothiocyanates and sulforaphane, which have been found to play roles in plant resistance to insect pests and pathogens (Hogge et al., 1988; Mithen et al., 1995; Benderoth et al., 2006; Hopkins et al., 2009). Aliphatic and indolic glucosinolates are the two most important types of glucosinolates present in the Brassicaceae family (Fahey et al., 2001; Mithen, 2001; Bekaert et al., 2012). Breakdown products of both aliphatic and indolic glucosinolates produced through hydrolysis by endogenous myrosinases (β-thioglucoside glucohydrolases) have anti-fungal properties in plants (Chew, 1988; Giamoustaris and Mithen, 1995; Manici et al., 1997; Agerbirk et al., 1998; Brader et al., 2001; Tierens et al., 2001; Barth and Jander, 2006; Stotz et al., 2011; Calmes et al., 2015).

The relationship between glucosinolate levels and the resistance of Brassicaceae family members to diverse fungal pathogens remains unclear. Upon *L. maculans* infection, overall levels of glucosinolates are not strongly correlated with pathogen resistance in different *Brassica* species (Mithen and Magrath, 1992; Sexton et al., 1999). In fact, a negative correlation between *Alternaria* infection and glucosinolate levels was reported in *Brassica napus* (Doughty et al., 1991; Giamoustaris and Mithen, 1997). By contrast, upon *Sclerotinia sclerotiorum* infection, the extent of pathogen-induced accumulation of indolic glucosinolates in *Brassica napus* is positively correlated with plant resistance (Li et al., 1999). These seeming contradictions could reflect the lifestyles of the individual fungal pathogens, e.g., necrotrophic versus biotrophic (Sanchez-Vallet et al., 2010), their host ranges, e.g., Brassicaceae-specialist versus broad-spectrum (Buxdorf et al., 2013), the genetic purity of the host plants, e.g., isogenic versus heterozygous lines, in addition to the range and quantity of glucosinolates and their break-down products produced by the host plants.

The association between total glucosinolate content and plant resistance to *L. maculans* infection has been studied since the 1990's. Nevertheless, no obvious relationship between glucosinolate profiles and *L. maculans* resistance has been established, possibly due to the fact that these studies focused on total glucosinolate levels rather than individual glucosinolate compounds (Mithen and Magrath, 1992; Giamoustaris and Mithen, 1997; Sexton et al., 1999; Kliebenstein et al., 2002). Previous studies concluded that the glucosinolate-myrosinase system is not a major determinant of blackleg resistance (Wretblad and Dixelius, 2000; Andreasson et al., 2001). This conclusion was supported by an earlier report in resynthesized *B. napus* F_2_ progeny, in which disease resistance and glucosinolate profiles did not co-segregate (Mithen and Magrath, 1992). By contrast, infection with the hemibiotrophic fungus *L. maculans* was shown to trigger the accumulation of both aliphatic (gluconapin, i.e., 3-butenyl glucosinolate; progoitrin, i.e., 4-hydroxybutyl glucosinolate; glucobrassicanapin, i.e., 4-pentenyl glucosinolate; and gluconapoleiferin, i.e., 2-hydroxy-4-pentenyl glucosinolate) and indolic (glucobrassicin, i.e., 3-indolylmethyl glucosinolate and 4-hydroxy-glucobrassicin) glucosinolates in *Brassica rapa* (Abdel-Farid et al., 2010). A few recent reports have shown that resistance to obligate biotrophs, hemibiotrophs, and necrotrophs might be associated with the production of indolic glucosinolates in Brassicaceae (Bednarek et al., 2009; Hiruma et al., 2013). In Arabidopsis, accumulation of indolic 4-methoxy-glucobrassicin in response to fungal attack was regulated by the expression of *CYP81F2*, which is activated by the myrosinase PEN2 (Bednarek et al., 2009). These contrasting reports on the association of glucosinolate profiles and resistance in *Brassica* species call for further investigation of plant-pathogen interactions at the molecular and biochemical levels.

Our previous studies have shown that the upregulation of glucosinolate biosynthesis genes is associated with the upregulation of individual glucosinolate compounds in *B. oleracea* inbred lines (Robin et al., 2016; Yi et al., 2016). Here, we investigate changes in expression of genes associated with glucosinolate biosynthesis upon infection with *L. maculans* and correlate these changes with alterations in glucosinolate profiles. These experiments were performed in both resistant and susceptible genotypes against two different *L. maculans* isolates.

## Materials and methods

### Plant materials and growth conditions

**Figure 1:**
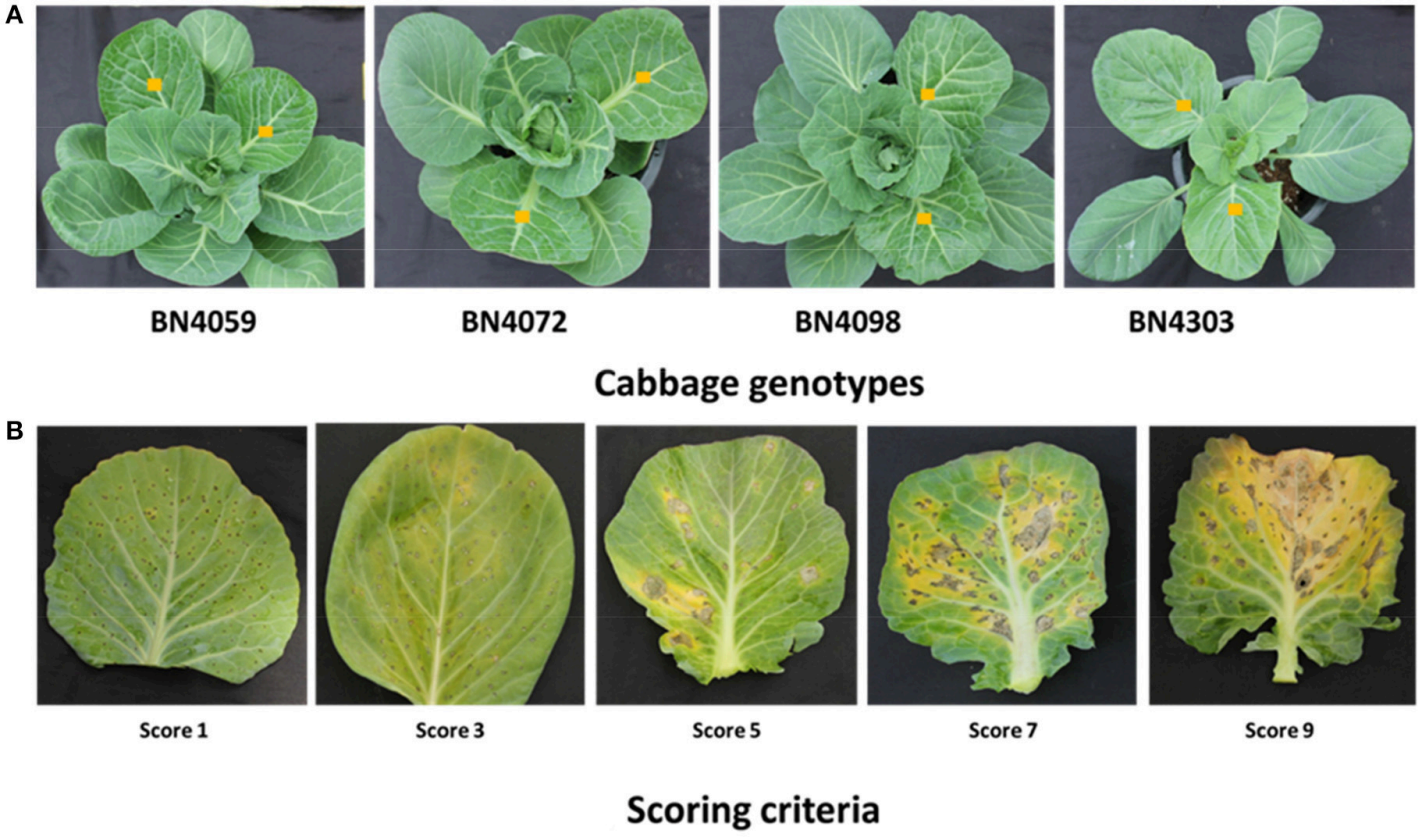
Cabbage lines **(A)** and scoring criteria **(B)** against blackleg disease. Three-month-old cabbage plants were inoculated to test their response to two *Leptosphaeria maculans* isolates 03–02 s and 00–100 s. Middle-aged leaves with yellow squares were infected. Infected leaves were scored two weeks after inoculation on a scale from 1−9 based on visual scoring criteria, where a score of “1” represents the least infected and “9” represents the most infected cabbage plants. In our previous assessment of seedling resistance, the lines BN4059 and BN4072 were susceptible while BN4098 and BN4303 were resistant (Robin et al., 2017a).

Seeds of four cabbage inbred lines (*B. oleracea* var. *capitata*) were obtained from Asia Seeds Ltd. (Seoul, South Korea). Two of the lines were reported to be resistant to blackleg disease at the seedling stage and the other two were susceptible (Figure 1; Robin et al., 2017a). Seeds were sown in a garden-soil mixture composed of peat moss, coco peat, perlite, zeolite, and vermiculite in 32-celled trays in a plant culture room with a temperature of 20 ± 2°C, 16-h day-length, and a light intensity of ca. 400 μmol m^−2^ s^−1^ at bench level (florescent light bulbs; Yi et al., 2015). Seedlings were grown for four weeks before transfer to a glasshouse, where they were grown for another two months before infection with *L. maculans*.

### *L. maculans* isolates and inoculation

Details of the two fungal isolates, culture methods, and spore solution preparation have been previously published (Robin et al., 2017a). In brief, two isolates of *L. maculans*, 03-02s (*AvrLm1-4-6-7-11-J1-S, AvrLep1-2-3*) and 00-100s (*AvrLm2-3-6-9-J1-S, AvrLep1-2*), were provided by Agriculture and Agri-Foods (AAFC), Saskatoon, Canada. Each isolate was sub-cultured on 20% V8 agar plates and grown at 22°C with a 16-h photoperiod under fluorescent light to produce a fungal inoculum. After 10 days, each plate was flooded with 10 mL of sterile distilled water and scraped with a sterile microscope slide to prepare a spore suspension. The spore suspension was filtered using sterile Miracloth (EMD Millipore Corporation, USA) to remove the mycelia and other debris. The clear suspension was centrifuged to concentrate the spores. The spore suspensions of 03–02 s and 00–100 s isolates were then diluted to 2.25 × 10^7^ spores mL^−1^ and used to inoculate small wounds created on middle-aged leaves of 3-month-old cabbage plants (Figure 1A). The experiment was laid out using a randomized block design with six plants per isolate. Approximately four wounds were created per cm^2^ leaf area. A set of six plants were kept as controls and another six plants were wounded but not infected (mock-treated). Diseased areas on the leaf surface were scored two weeks after inoculation on a scale of 1–9, based on the severity of the infection (Figure 1B), and categorized as resistant (R, score range 1.0–3.0), moderately resistant (MR, 3.1–6.0), susceptible (S, 6.1–7.0) and highly susceptible (HS, 7.1–9.0).

### Collection and preparation of leaf samples for HPLC and expression analysis

Leaf samples from three biological replicates, randomly chosen from six inoculated plants, were collected from control, mock-treated, and *L. maculans* inoculated plants four days after inoculation to estimate endogenous glucosinolate content and quantify the expression of selected genes from the glucosinolate biosynthesis pathway (Figure S1). Leaf samples collected for HPLC analysis and real-time PCR were immediately stored at −80°C after flash-freezing in liquid nitrogen.

### Estimation of glucosinolate content

Leaf samples from three biological replicates from each of the control, mock, and *L. maculans* infected plants were used for extracting desulfo-glucosinolates through a modified HPLC protocol as previously described (Yi et al., 2015, 2016; Robin et al., 2016). Frozen leaf tissue stored at −80°C was treated with methanol and then ground to a fine powder. The powdered leaf samples were preserved at 70°C for 10 min and then kept at room temperature for about an hour. The powdered samples were then centrifuged at 10,000 × *g* at 4°C for 8 min. This centrifugation step removes structural components and protein molecules as sediments. The supernatant was then passed over an anion-exchange column. Centrifugation and anion-exchange chromatography was repeated two more times. The supernatant collected from the final step of anion-exchange chromatography was considered the crude glucosinolate sample. The crude glucosinolates were then subjected to desulfation. Here, 0.5 mL 50 mM barium acetate and 0.5 mL 50 mM lead acetate were mixed with the crude glucosinolates followed by centrifugation at 2,000 × *g* for 10 min. The supernatant was then passed through a pre-equilibrated (with 0.5 M sodium acetate) DEAE-Sephadex column. Desulfation was initiated by the addition of 250 μL aryl sulfatase to the column and was allowed to run over-night for 16 h. The desulfated glucosinolates were then eluted with 1 mL distilled water. The eluted desulfo-glucosinolates were purified by high-speed centrifugation at 20,000 × *g* for 4 min at 4°C followed by filtering through a PTFE filter (13 mm, 0.2 μm, Advantec, Pleasanton, CA, USA). The purified glucosinolates were then subjected to HPLC analysis in a Waters 2695 HPLC system (Waters, Milford, MA, USA) equipped with a C_18_ column (Zorbax Eclipse XBD C_18_, 4.6 mm × 150 mm, Agilent Technologies, Palo Alto, CA, USA). Both water and acetonitrile were used as mobile phase solvents. Individual glucosinolate compounds were detected using a PDA 996 UV-visible detector (Waters) at a wavelength of 229 nm. For quantification of the detected glucosinolates, a standard curve prepared from commercial sinigrin (SIN) was used. Mass spectrometry analysis (HPLC/MS, Agilent 1,200 series, Agilent Technologies) facilitated the identification of individual glucosinolates (Yi et al., 2016). Chemical names, common names, abbreviations, and chemical structures of the glucosinolates identified in this study are given in Figure S2.

### Primer design for expression analysis of glucosinolate biosynthesis genes

Thirty-eight genes involved in glucosinolate biosynthesis were selected for transcription analysis to determine how transcript levels are affected by pathogen inoculation (Table S1, Figure S1). Eleven of the genes encode transcription factors: five in the aliphatic biosynthesis pathway and six in the indolic pathway. Of the other 27 genes, 10 are aliphatic biosynthesis genes and 17 are indolic biosynthesis genes (Robin et al., 2016; Yi et al., 2016). Primers were designed using primer3plus software (http://primer3plus.com/) and primer efficiency was tested according to Robin et al. (2016).

### cDNA synthesis and real-time quantitative PCR analysis

Total RNA was extracted from the collected leaf samples using RNeasy mini kit, Catalog No. 74106, Qiagen, Valencia, CA, USA. cDNA synthesis was performed from total RNA using a PrimeScript-based kit (Takara Bio, Inc., Shiga, Japan). To conduct quantitative RT-PCR (qPCR), iTaqTM SYBR® Green Super-mix was used with ROX (Bio-Rad, Hercules, CA, USA). For each reaction, a total reaction volume of 20 μL was prepared containing 10 μL PCR master mix, 7 μL ultra-pure water, 2 μL forward and reverse primers and 1 μL cDNA template with a concentration of 60 ng μL^−1^. PCR conditions were as follows: denaturation at 95°C for 10 min, 40 cycles of amplification with denaturation at 95°C for 20 s, annealing at 58°C for 20 s, and amplification and signal acquisition at 72°C for 30 s. Data were recorded as fluorescence at the end of each of 40 cycles for each sample. Each biological replicate was tested in three technical replicates. Quantification (Cq) analysis was done using LightCycler96 software (Roche, Mannheim, Germany). Livak's comparative 2^−ΔΔ*Ct*^ method was used to calculate the relative expression of each sample (Livak and Schmittgen, 2001). Three different *actin* genes selected from the NCBI database, GenBank Accession Nos. AF044573 (Zhang et al., 2012), JQ435879 (Nawaz et al., 2014), and XM_013753106 (Lee et al., 2015), were expressed in all inbred lines and were used as a reference.

### Statistical analysis

A one-way analysis of variance was conducted to test the statistical significance of different treatments on the four cabbage inbred lines using Minitab 18 statistical software (Minitab Inc., State College, PA, USA). A *posthoc* Tukey's pairwise comparison was conducted to visualize statistical significance of 16 treatment × genotype combinations. Test statistic, degrees of freedom, and p-values of statistical significance for glucosinolate contents and relative expression of biosynthesis genes are given in Table S2.

Separate heat maps were drawn to show association between blackleg scores and glucosinolate contents or expression of genes using conditional formatting option in Microsoft Excel. Pearson correlation coefficient was estimated between glucosinolate content and expression of biosynthesis genes in 16 treatment × genotype combinations (Supplementary Appendix 1).

## Results

### Resistance of adult cabbage lines to *L. maculans* infection

Inoculation of cabbage leaves with two *L. maculans* isolates resulted in different responses in different lines, based on visual scoring of blackleg disease symptoms (Table 1). Of the eight total combinations of four cabbage genotypes and two isolates (as shown in Table 1) only one combination, BN4303 × 00-100s, exhibited complete resistance, with the lowest visual score of 2 (range 1–3). The combinations of BN4303 × 03–02 s (score range 3–5) and BN4098 × 00–100s (score range 4–6) showed moderate resistance. BN4098, which exhibited moderate resistance to isolate 00–100 s, was susceptible to isolate 03–02 s (score range 6–8). Lines BN4072 and BN4059 were susceptible to both isolates, with the latter being highly susceptible to both isolates (Table 1). Overall, line BN4303 was the most resistant, as it showed complete resistance to isolate 00–100 s and moderate resistance to 03–02 s, whereas BN4098 showed moderate resistance only to isolate 00–100 s and the other two lines were susceptible.

**Table 1:** Resistance scoring of four cabbage lines at three months of age (adult stage) against two *L. maculans* isolates “03–02 s” and “00–100 s” 15-days post-inoculation.

**Line**	**Seedling resistance status against both isolates**	***L. maculans*** **isolates**					
		**03–02 s**			**00–100 s**		
		**Score**	**Score range**	**Interaction**	**Score**	**Score range**	**Interaction**
BN4059	HS	9.0	7–9	HS	8.0	6–9	HS
BN4072	HS	7.0	6–8	S	7.0	7–9	S
BN4098	R	7.0	6–8	S	5.0	4–6	MR
BN4303	R	4.0	3–5	MR	2.0	1–3	R

*Each score is the median of six observations. R, Resistant; MR, moderately resistant; S, susceptible and HS, highly susceptible*.

### Constitutive glucosinolate contents vary in cabbage inbred lines

**Figure 2:**
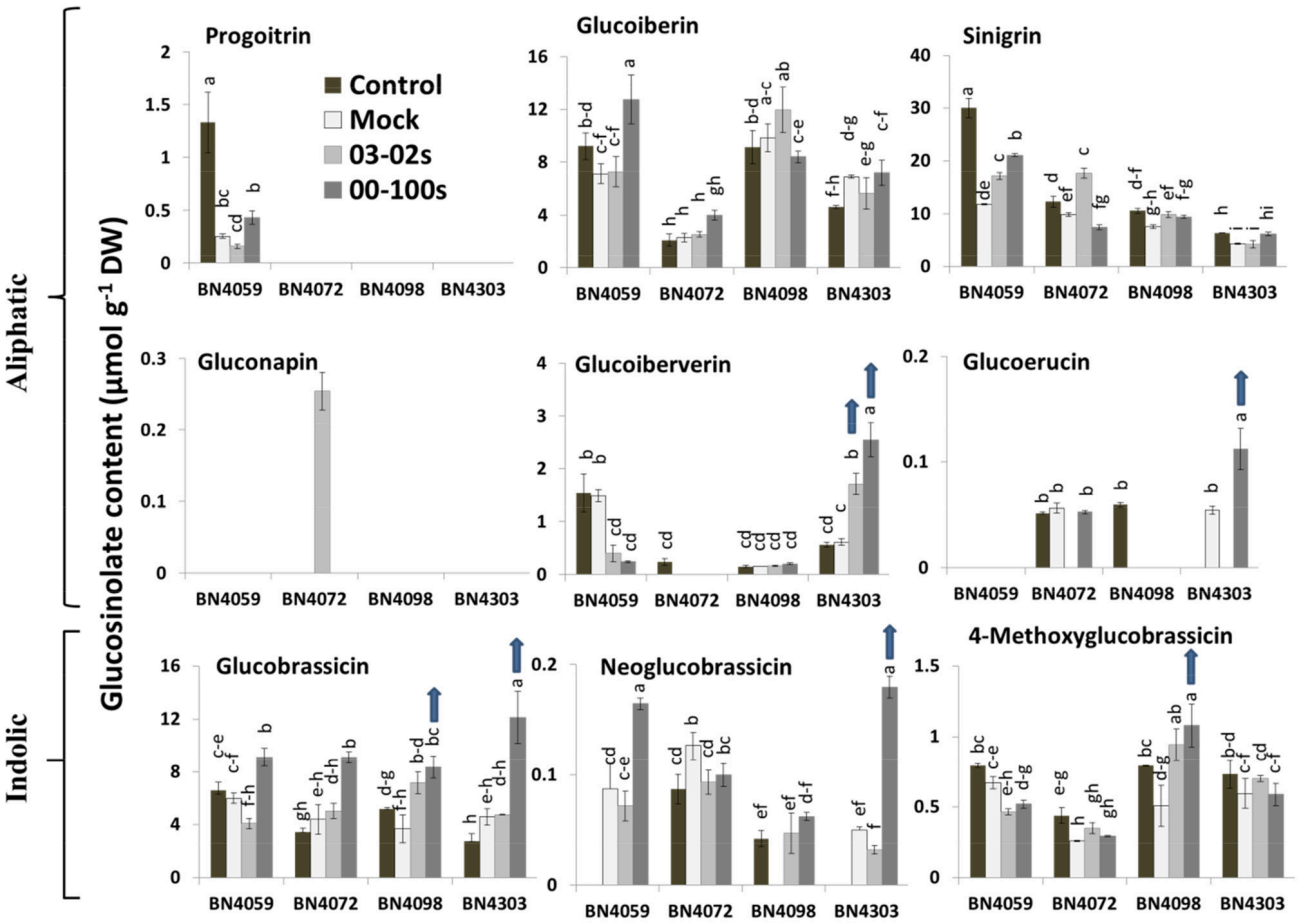
Glucosinolate contents of leaf samples from the four cabbage lines (BN4059, BN4072, BN4098, and BN4303) under four different treatments (control, mock, inoculation with 03–02 s or 00–100 s) four days after inoculation. Detection limit of glucosinolates was 0.025 μmol g^−1^ DW. The mean of three biological replicates is presented. Vertical bars indicate standard error of mean. Different letters indicate statistically significant differences between genotype × treatment combinations. Upward-pointing blue arrows indicate increased glucosinolate content in response to *L. maculans* infection.

In untreated control plants, levels of both aliphatic and indolic glucosinolates varied significantly between the four cabbage lines (Figure 2, Supplementary excel data file). Among the control plants, BN4059, the most susceptible cabbage line, had the highest total levels of glucosinolates while BN4303, the most resistant line, had the lowest total levels (Figure S3). PRO was found only in BN4059, which also had the highest levels of aliphatic glucoiberin (GIB), sinigrin (SIN), and glucoiberverin (GIV), but no glucoerucin (GER). BN4059 also had the highest levels of indolic GBS and MGBS, but no NGBS. Notably, BN4303 had the lowest levels of SIN between the four lines. Hydroxy-glucobrassicin (HGBS), another indolic glucosinolate, was detected only in untreated control plants of the BN4098 line (Figure S3).

### *L. maculans* inoculation alters the glucosinolate profile of adult cabbage plants

Inoculation of 3-month-old cabbage plants with either of the two *L. maculans* fungal isolates remarkably changed the glucosinolate profiles in leaves of the fully and moderately resistant cabbage lines. The content of aliphatic GIV increased significantly in BN4303 in response to both fungal isolates while it decreased or was not produced in the susceptible genotypes BN4059 and BN4072 (Figure 2). In the resistant line BN4303, levels of GIV increased by 2.77- and 4.14-fold four days after inoculation with 03–02 s and 00–100 s, respectively compared to mock-treated plants (Figure 2). By contrast, in the most susceptible line BN4059, GIV levels decreased 3.72- and 6.28-fold following fungal inoculation with 03-02s and 00-100s, respectively, compared to mock-treated plants (Figure 2).

Infection with 00–100 s induced the accumulation of glucoerucin (GER) in the resistant line BN4303; no GER was detected in control plants (Figure 2). The most susceptible line, BN4059, did not produce any glucoerucin (GER). Other notable changes in aliphatic glucosinolates were (i) a 3- to 8-fold decrease in progoitrin (PRO) levels in mock-treated and fungal-inoculated plants in the susceptible cabbage line BN4059 compared to the untreated plants, indicating that PRO levels are sensitive to both leaf injury and fungal attack; (ii) accumulation of gluconapin (GNA) in BN4072 in response to 03–02 s infection and (iii) an increase in GER in mock-treated BN4303 plants compared to the untreated plants, indicating that GER levels are also sensitive to both leaf injury and fungal attack (as GER levels further increased in the same line upon 00–100 inoculation).

After inoculation of BN4303 with 00–100 s, levels of glucobrassicin (GBS) and neoglucobrassicin (NGBS) increased by 2.65- and 3.64-fold, respectively, compared to the mock-treated plants (Figure 2). Upon infection of the moderately resistant line BN4098 with 00–100 s, levels of GBS and methoxy-glucobrassicin (MGBS) increased by 2.28- and 2.13-fold compared to the mock-treated plants, respectively (Figure 2).

In contrast to the resistance-related upregulation, indolic GBS content was increased in two susceptible interactions, BN4059 × 00-100s (1.52-fold) and BN4072 × 00-100s (2.06-fold), compared to the mock-treated plants (Figure 2). NGBS content was also increased in BN4059 × 00–100s (1.89-fold) compared to the mock-treated plants. In addition to GBS and NGBS, the aliphatic GIB content also increased 1.79-fold in BN4059 × 00–100 s interaction compared to the mock-treated plants (Figure 2).

Overall, our results show that the complete resistance of the line BN4303 against 00–100s isolate was associated with simultaneously increased contents of both aliphatic (GIV and GER) and indolic (GBS and NGBS) glucosinolates upon inoculation, while the enhanced accumulation of an aliphatic glucosinolate (GIV) was associated with moderate resistance in this line against 03–02 s isolate (Table 2). By contrast, the moderate resistance of the line BN4098 against the 00–100 s isolate was associated with enhanced accumulation of only indolic glucosinolates (GBS, NGBS and MGBS).

**Table 2:** Association between increased glucosinolate contents and increased expression of glucosinolate pathway genes in resistant and moderately resistant disease interactions.

	**BN4098 × 00–100 s (moderately resistant interaction)**	**BN4303 × 03–02 s (moderately resistant interaction)**	**BN4303 × 00–100 s (resistant interaction)**
Aliphatic	—	GIV	GIV and GER
		(*ST5b*–*Bol026201, Bol026202*)	(*ST5b*–*Bol026201, Bol026202*)
			*GSL-OH*
			(*Bol033373*)
Indolic	GBS	—	GBS
	(*ST5a*-*Bol026200*)		(*MYB34*-*Bol007760, ST5a*-*Bol026200*)
	MGBS		NGBS
	(*CYP81F4*-*Bol032712, CYP81F2*-*Bol026044*)		(*CYP81F4*-*Bol032712, Bol032714*; *CYP81F2*-*Bol026044*)

*GIV, Glucoiberverin, GER, glucoerucin, GBS, glucobrassicin, NGBS, neoglucobrassicin and MGBS, methoxy-glucobrassicin*.

### Expression changes in transcription factor and aliphatic glucosinolate biosynthesis genes in cabbage lines with blackleg disease

**Figure 3:**
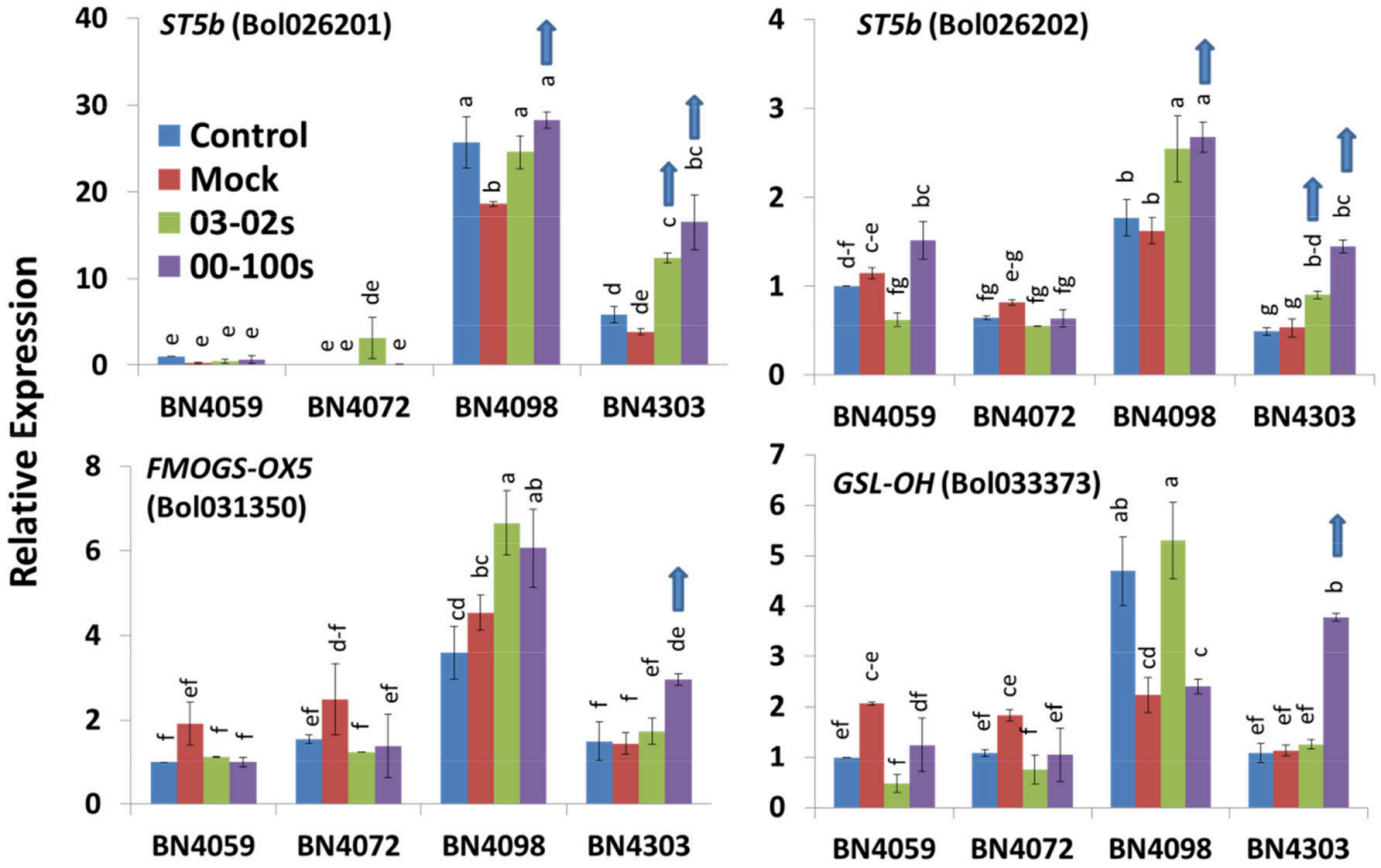
Relative expression of aliphatic glucosinolate biosynthesis genes in leaf samples from the four cabbage lines (BN4059, BN4072, BN4098, and BN4303) under four different treatments (control, mock, inoculation with 03–02 s or 00–100 s) four days after inoculation. The mean of three biological replicates is presented. Vertical bars indicate standard error. Different letters indicate statistically significant differences between genotype × treatment combinations. Upward-pointing blue arrows indicate increased glucosinolate content in response to *L. maculans* infection.

The expression levels of majority of the transcription factors under untreated control conditions were comparatively higher in BN4098 compared to the other three lines (Figure S4, Supplementary excel data file). Like the transcription factor genes, the expression levels of most of the aliphatic structural biosynthesis genes were comparatively higher in BN4098 than in the other three cabbage lines under untreated control conditions (Figure 3, Figure S5). However, FMOGS-OX2 (Bol010983) and FMOGS-OX5 (Bol029100) showed higher expression in BN4059 control plants compared to the other three lines, consistent with the highest content of total, and most individual, aliphatic glucosinolates in this genotype (Figure S5).

None of the genes for aliphatic transcription factors were upregulated in any resistance combination; rather, expression of these five genes generally decreased in this genotype upon infection (Figure S4). Expression of *ST5b* and *GSL-OH*, which are involved in the biosynthesis of aliphatic glucosinolates, increased in both of the resistant lines, BN4303 and BN4098, during blackleg infection (Figure 3). In the resistant line BN4303, expression of *ST5b* genes *Bol026201* and *Bol026202* increased, respectively, 4.31- and 2.73-fold after 00–100 s infection and 3.23- and 1.70-fold after 03–02 s infection compared to mock-treated plants (Figure 3). Expression of *GSL-OH* increased 3.33-fold in BN4303 after 00–100s infection compared to mock-treated plants. In susceptible combinations, BN4072 × 03-02s and BN4098 × 03-02s, *AOP2* (*Bo2g102190*) showed 24.4- and 3.39-fold higher expression, respectively, compared to mock treated plants (Figure S5). *AOP2* also showed a 2.7-fold increase in BN4303 × 03–02 s compared to mock treated plants (Figure S5).

### Expression changes in transcription factors involved in the biosynthesis of indolic glucosinolates in cabbage lines with blackleg disease

**Figure 4:**
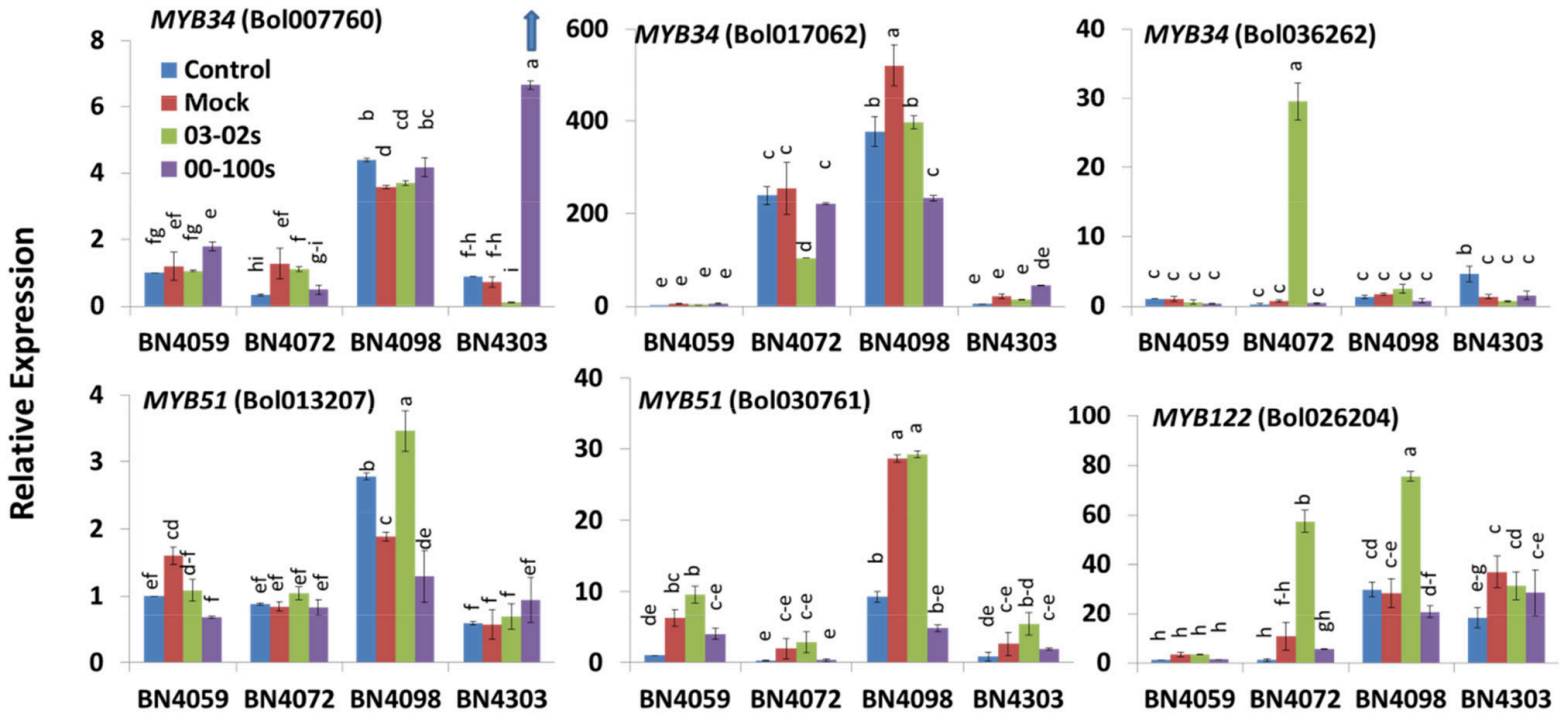
Relative expression of indolic glucosinolate transcription factor related genes in leaf samples from the four cabbage lines (BN4059, BN4072, BN4098, and BN4303) under four different treatments (control, mock, inoculation with 03–02 s or 00–100 s) four days after inoculation. The mean of three biological replicates is presented. Vertical bars indicate standard error. Different letters indicate statistically significant differences between genotype × treatment combinations. Upward-pointing blue arrows indicate increased glucosinolate content in response to *L. maculans* infection.

The expression levels of five *B. oleracea* genes encoding three transcription factors were measured under control, mock, and infection conditions. In untreated control plants, five out of six transcription factors involved in indolic glucosinolate biosynthesis, the exception being *MYB34* (*Bol036262*), were highly expressed in BN4098 compared to the other three lines (Figure 4). The genes *Bol007760* and *Bol017062*, encoding the transcription factor *MYB34*, showed increased expression in the resistant line BN4303 with blackleg disease. *Bol007760* and *Bol017062* showed a 9.35- and 2.13-fold increase against 00–100s isolates, respectively, compared to mock-treated plants (Figure 4). By contrast, *MYB34* (*Bol036262*), and *MYB122* (*Bol026204*) showed a 44.1- and 5.49-fold increase, respectively, in the susceptible line BN4072 under 03–02 s infection compared to mock-treated plants (Figure 4).

### Expression changes in indolic glucosinolate biosynthesis genes in cabbage lines with blackleg disease

**Figure 5:**
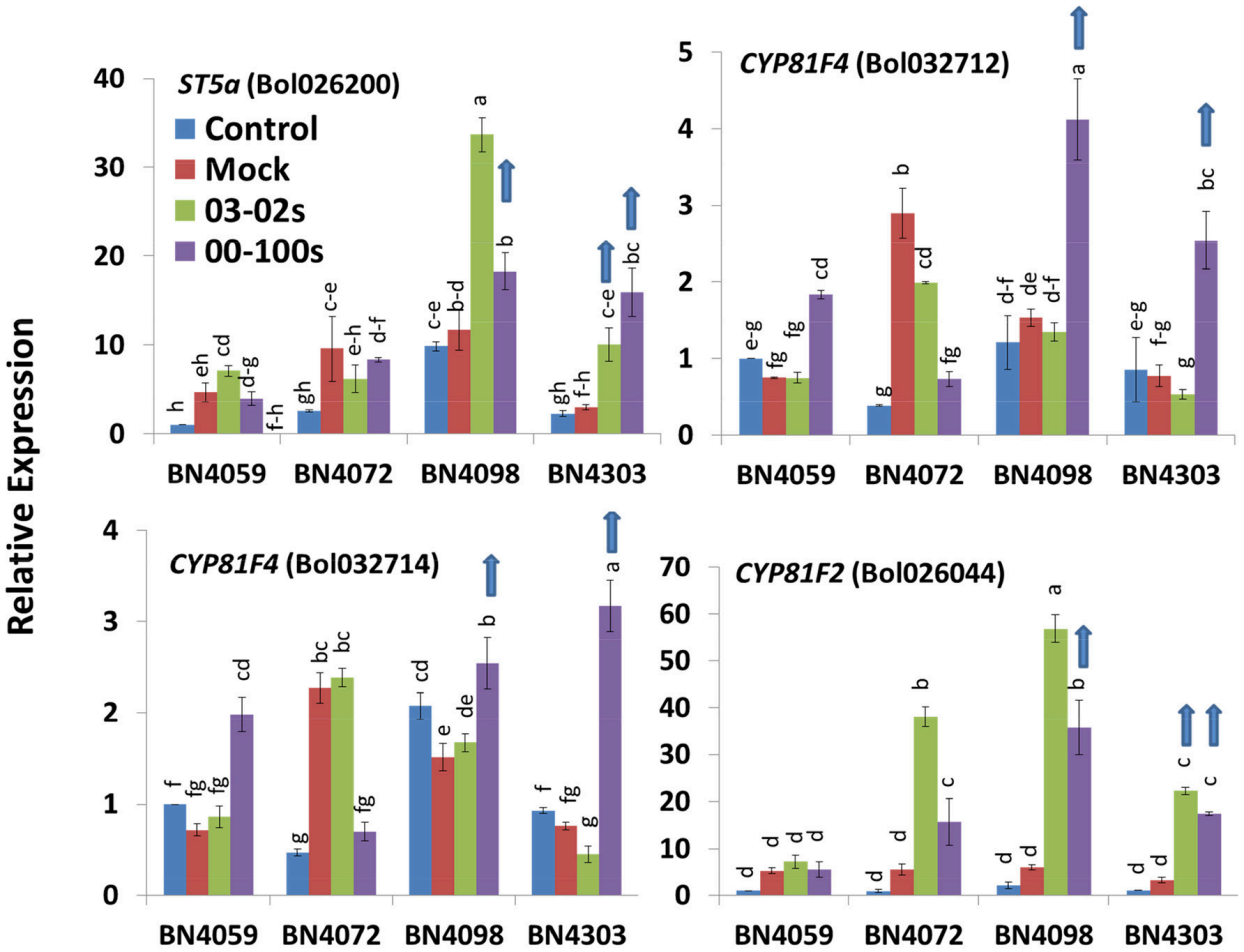
Relative expression of indolic glucosinolate biosynthesis genes in leaf samples from the four cabbage lines (BN4059, BN4072, BN4098, and BN4303) under four different treatments (control, mock, inoculation with 03–02 s or 00–100 s) four days after inoculation. The mean of three biological replicates is presented. Vertical bars indicate standard error. Different letters indicate statistically significant differences between genotype × treatment combinations. Upward-pointing blue arrows indicate increased glucosinolate content in response to *L. maculans* infection.

In untreated control plants, two genes, *CYP81F2* (*Bol014239*) and *CYP81F3* (*Bol028919*), showed higher expression in BN4303 than in the other three lines. Five genes including *CYP81F1* (*Bol017375*), *CYP81F1* (*Bol017376*), *CYP81F1* (*Bol028914*), *CYP81F2* (*Bol012237*), and *IGMT1* (*Bol020663*) showed higher expression in BN4072, which had higher NGBS contents than the other lines. In addition, ten genes showed higher expression in BN4098, in which the corresponding MGBS content was higher compared to the other three cabbage lines (Figure 5, Figure S6).

*ST5a* (*Bol026200*), *CYP81F4* (*Bol032712, Bol032714*), and *CYP81F2* (*Bol026044*), which are associated with indolic glucosinolate biosynthesis, were upregulated in resistant cabbage lines upon infection. *ST5a* (*Bol026200*) exhibited a 5.31- and 3.36-fold increase, respectively, in BN4303 in response to 00–100 s and 03–02 s compared to mock-treated plants (Figure 5, Figure S6). This gene also showed a 1.28-fold increase in BN4098 under 00–100s infection compared to the mock-treated plants (Figure 5). *ST5a* (*Bol039395*) showed 1.5- and 1.58-fold increased expression in BN4303 in response to 00–100 s and 03–02 s compared to mock-treated plants (Figure S6). *CYP81F4* (*Bol032712*) showed a 2.7- and 3.29-fold increase in BN4098 and BN4303, respectively, in response to 00–100 s compared to the mock-treated plants (Figure 5). *CYP81F4* (*Bol032714*) showed a 4.16-fold increase in BN4303 under 00–100s infection compared to the mock-treated plants. *CYP81F4* (*Bol028918*) showed 2.94-fold higher expression in BN4303 × 00–100 s compared to the mock-treated plants (Figure S6). *CYP81F3* (*Bol032711*) exhibited 2.05-, 2.47-, and 2.16-fold increases in expression in BN4098 × 00–100 s, BN4303 × 03–02 s and BN4303 × 00–100 s, respectively, compared to the mock-treated plants (Figure S6).

*CYP81F2* (*Bol026044*) showed a 6.78- and 5.31-fold increase in BN4303 under 03-02s and 00–100s infection, respectively, and also showed a 5.89-fold increase in BN4098 under 00–100 s infection compared to the mock-treated plants (Figure 5). *CYP81F2* (*Bol014239*) showed 8.05-fold higher expression in BN4098 × 00–100 s compared to the mock-treated plants (Figure S6). *CYP81F1* (*Bol028913*) displayed a 2.71- and 6.66-fold increase in BN4303 infected with 00–100s compared to the mock-treated plants (Figure S6).

Notably, increased expression of some indolic glucosinolate genes was also observed in susceptible plants. For example, *ST5a* (*Bol026200*) in the BN4098 × 03–02 s combination increased 3.24-fold, *CYP81F2* (*Bol026044*) increased 9.21-fold in the BN4098 × 03–02 s combination and 6.91-fold in the BN4072 × 03–02 s combination (Figure 5). *ST5a* (*Bol039395*), *CYP81F2* (*Bol014239*) and *IGMT1* (*Bol007029*) increased 2.56-, 33.55-, and 4.43-fold, respectively, in BN4098 infected with 03–02 s compared to the mock-treated plants (Figure S6).

### Association between pathogen-induced upregulation of pathway genes and accumulation of corresponding glucosinolates and the plant's resistance status

**Figure 6:**
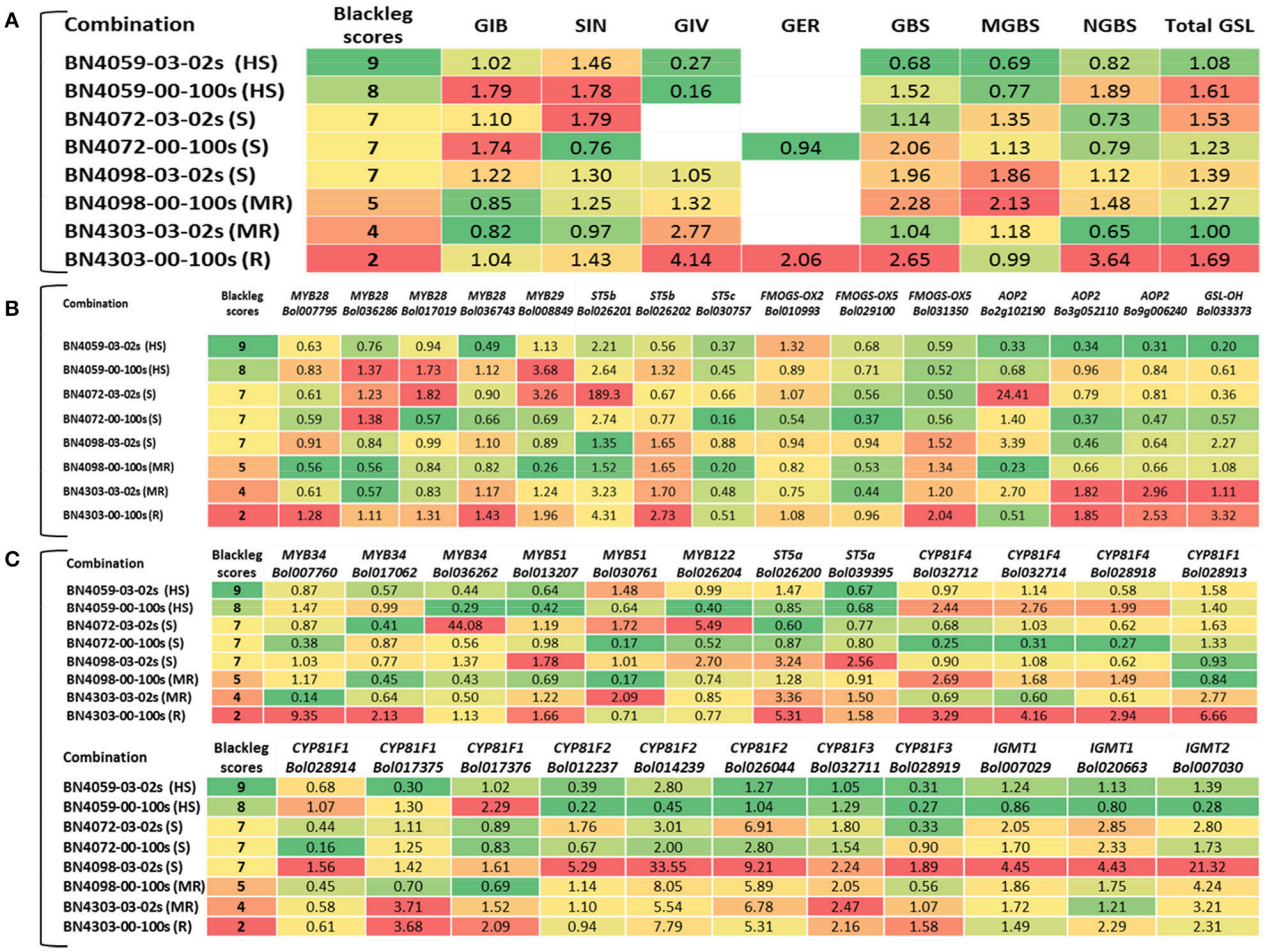
Simple grouping assay in heat maps showing comparison between blackleg scores and **(A)** fold changes in glucosinolate contents, **(B)** fold changes in expression of aliphatic glucosinolate biosynthesis genes and **(C)** fold changes in expression of indolic glucosinolate-biosynthesis related genes in *L. maculans*-inoculated leaf samples compared to respective mock-treated samples in HS, highly susceptible; S, susceptible; MR, moderately resistant and R, resistant combination. Red color represents blackleg disease resistance and increased accumulation of glucosinolates and expression of biosynthesis genes.

**Figure 7:**
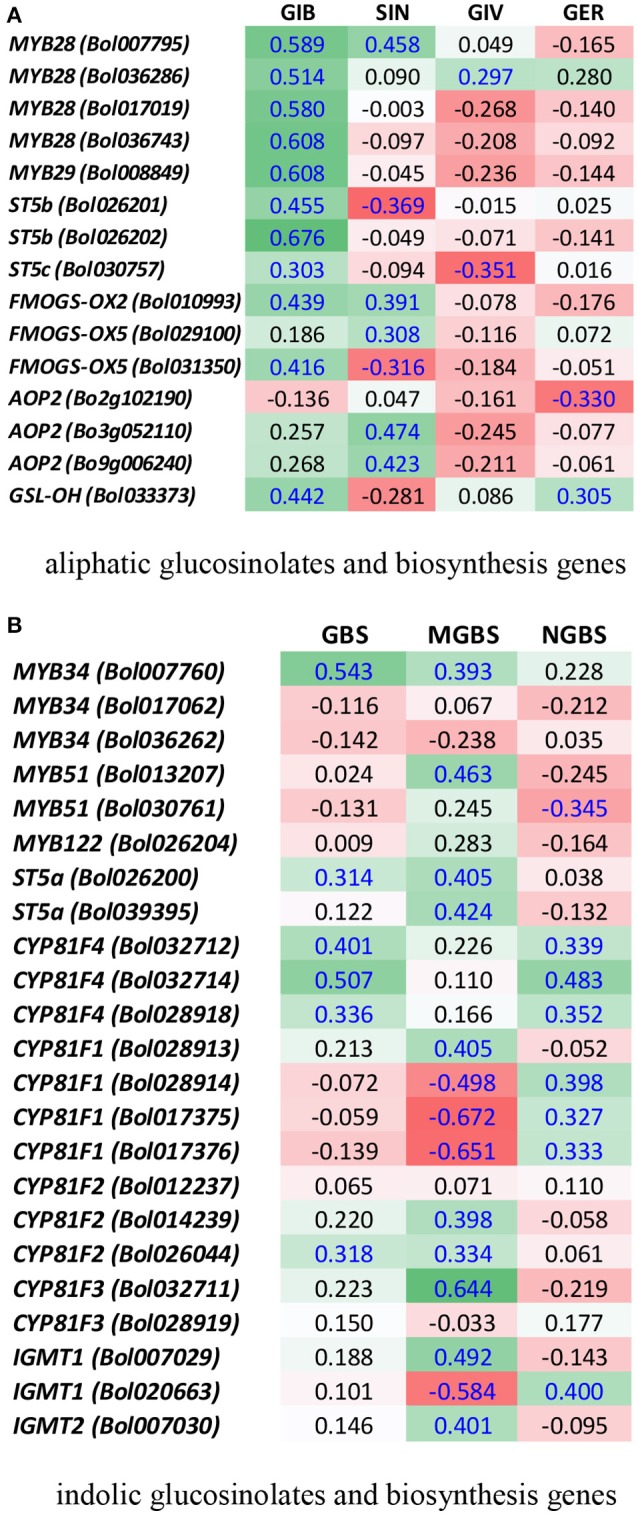
Heatmap showing correlation between the levels of aliphatic **(A)** and indolic **(B)** glucosinolates and expression of biosynthesis genes under four treatments in four cabbage lines. Blue letters represent statistically significant correlation (*p* < 0.05). For each gene and glucosinolate combination, the values indicate Pearson correlation coefficient. Green cells represent positive correlation and red cells represent negative correlation. GIB, Glucoiberin; SIN, sinigrin; GIV, glucoiberverin; GER, glucoerucin; GBS, glucobrassicin; NGBS, neoglucobrassicin; and MGBS, methoxyglucobrassicin.

A heat map emphasized that the fold changes under pathogen inoculation compared to mock-treated samples in expression of genes involved in the biosynthesis of glucosinolates were often consistent with the blackleg resistance in the cabbage lines. For example, in the resistant line BN4303 under 00–100 s infection when *ST5b* (*Bol026201* and *Bol026202*) and *GSL-OH* (*Bol033373*) expression was increased, the accumulation of two aliphatic glucosinolates GIV and GER was also increased (Figures 6A,B). Higher expression levels of the *ST5b* genes *Bol026201* and *Bol026202* were associated with an increased content of aliphatic GIV in the moderately resistant interaction of BN4303 × 03–02 s. However, Pearson correlation coefficients showed positive correlation only between GIV content and *MYB28 (Bol007795)* expression (Figure 7A). GER content exhibited a positive correlation with both *MYB28* (*Bol007795*) and *GSL-OH* (*Bol033373*). In the BN4303 × 00–100 s interaction, increased expression of *MYB34* (*Bol007760*) and *ST5a* (*Bol026200*) was concomitant with higher levels of GBS, and upregulation of *CYP81F4* (*Bol032712, Bol032714*) and *CYP81F2* (*Bol026044*) was associated with increased NGBS content (Figures 6A,C). Pearson correlation tests indicated a positive association of GBS content with both *MYB34* (*Bol007760*) and *ST5a* (*Bol026200*) expression along with that of *CYP81F4* (*Bol032712, Bol032714*), *CYP81F4 (Bol028918)*, and *CYP81F2* (*Bol026044*). NGBS content showed a positive correlation with *CYP81F4* (*Bol032712, Bol032714*) expression along with *CYP81F4 (Bol028918), CYP81F1*, and *IGMT1 (Bol020663)* expression (Figure 7B). In another moderately resistant interaction, BN4098 × 00–100 s, increased expression of *ST5a* (*Bol026200*) corresponded to an increased content of GBS (Figures 6A,C). Furthermore, the increased expression of *CYP81F4* (*Bol032712, Bol032714*) and *CYP81F2* (*Bol026044*) corresponded with an increased content of MGBS. Among these three genes, MGBS content showed positive correlation only with *CYP81F2* (*Bol026044*). In the susceptible interaction BN4059 × 00–100 s with increased content of GBS, expression of *CYP81F4* genes was also increased (Figures 6A,C, 7B). These data demonstrate a connection between these genes and changes in the glucosinolate profiles of resistant plants in response to *L. maculans* infection although a few deviations are often noted.

## Discussion

### Resistance of cabbage inbred lines differs between the seedling and adult stages

It has long been known that blackleg resistance in adult plants could be different from that in seedlings, as resistance at these stages has been reported to be under differing genetic control (Ballinger and Salisbury, 1996; Larkan et al., 2016). It was also reported that adult plant resistance is not race-specific (Rimmer, 2006), a conclusion that was later debated by other studies (e.g., Marcroft et al., 2012; Raman et al., 2012). Despite our previous findings that line BN4098 is resistant to both 03–02 s and 00–100 s isolates at the seedling stage (Robin et al., 2017a), our current results show that is susceptible to 03–02 s at 3 months of age (Table 1). This difference in susceptibility–both at different growth stages and against different isolates of *L. maculans*–could be under genetic control, and will be a subject for further investigation.

### The aliphatic glucosinolates GIV and GER and the indolic glucosinolates GBS and NGBS are associated with resistance to blackleg disease

Associations between pathogen induced glucosinolate accumulation and plant resistance were detectable for certain combination of compounds only under particular line × pathovar combinations (Figure 6B). There were both positive and negative associations between glucosinolate abundance and resistance phenotypes. In addition, glucosinolate levels were often substantially altered under mock treatment compared to control plants. For instance, PRO and SIN contents were generally decreased in mock-treated plants compared to control plants in different line × pathovar combinations (Figure 2). However, the GIB, GIV, and GBS contents generally remained unaffected under mock treatment in different line × pathovar combinations compared to the control. We, therefore, considered glucosinolate level at the mock-treated samples as a benchmark to calculate pathogen-induced increase in accumulation. The contents of GBS and NGBS also exhibited upregulation in susceptible interactions in BN4059 and BN4072 (Figure 2). Aliphatic GIB and SIN contents were increased in BN4059 × 00–100 s combinations (Figure 2). Nevertheless, both aliphatic (glucoiberverin, glucoerucin) and indolic (glucobrassicin and neoglucobrassicin) glucosinolates increased to higher concentrations in the completely resistant line BN4303 upon blackleg infection (00–100 s isolate) (Table 2), indicating that resistance in adult cabbage plants may be achieved through an accumulation of both aliphatic and indolic glucosinolate compounds. These findings are somewhat different from current data that suggest indolic glucosinolates alone may provide resistance to nectrotrophic and hemibiotrophic pathogens (Sanchez-Vallet et al., 2010; Hiruma et al., 2013; Frerigmann et al., 2016; Wu et al., 2016). The absence of a pathogen-induced increase in either aliphatic or indolic glucosinolates was associated with only moderate resistance in the BN4098 × 00–100 s and BN4303 × 03–02 s combinations, respectively (Table 2). By contrast, susceptible interactions exhibited an increase in one or only a few aliphatic or indolic glucosinolate compounds with a simultaneous decrease in other compounds. During infection of BN4059 for example, there was a significant increase of GIB, SIN, GBS, and NGBS but a decrease in aliphatic GIV and indolic MGBS, which may have resulted in this line's susceptibility to 00–100s (Figure 2). Furthermore, the marked upregulation of a single glucosinolate was also not associated with resistance. For example, GNA increased dramatically in BN4072 upon infection with 03–02 s, but neither aliphatic GIV nor any of the three indolic glucosinolates were induced, which may have resulted in this line's susceptibility to this isolate (Figure 2). These data suggest that specific glucosinolate profiles may confer resistance to cabbage to *L. maculans* infection in a genotype- and isolate-specific manner.

### Increased expression of *St5b* genes led to increased levels of GER and GIV in resistant cabbage

*ST5b* genes are involved in secondary modifications of desulfo-glucosinolates to GIB and GIV and then to other aliphatic glucosinolate compounds (Figure S1, Liu et al., 2014; Yi et al., 2015). In a recent study, increased expression of *ST5b* genes was found to be associated with higher levels of three aliphatic glucosinolates, glucoraphanin, SIN, and GNA, which accumulated via GIB and GIV biosynthesis (Robin et al., 2016). In this study, infection of BN4303 with either 00–100 s or 03–02 s induced the expression of two *ST5b* genes, *Bol026201* and *Bol026202*; infected plants also showed a striking increase in GIV and GER levels (Figure 6A). Thus, our results indicate that infection by the blackleg pathogen induced the upregulation of these genes, subsequently leading to the increase in GIV that was associated with the resistance of plants (Figures 2, 3). However, this correlation based association is required to be validated further through genetic studies.

### GIV is a candidate to play a crucial role in blackleg disease resistance

The conspicuous contrasting patterns of GIV accumulation between the most resistant combination BN4303 × 00–100 s, which showed high levels of GIV, and the most susceptible combinations BN4059 × 00–100 s and BN4059 × 03–02 s, which showed low levels of GIV, highlight GIV as a potentially important contributor to glucosinolate-mediated resistance in adult cabbage plants (Figure 2). Moreover, a decrease in GIV levels upon infection in the two most susceptible combinations BN4059 × 03–02 s and BN4059 × 00–100 s further indicated that lower GIV levels are associated with reduced plant resistance against blackleg infection (Figure 2). By contrast, in the modestly resistant combinations BN4098 × 00–100 s and BN4303 × 03–02 s, GIV was completely absent and highly upregulated, respectively. This discrepancy indicates that GIV cannot provide full resistance alone, but rather is likely to be a contributor to resistance, possibly together with indolic GBS and NGBS (Figure 2). A higher accumulation of GIV coupled with GBS and NGBS was also found to show a positive association with lower feeding scores as reflected by less leaf area damage by the Diamondback moth in cabbage (Robin et al., 2017b).

### *MYB34* likely activates glucosinolate biosynthesis genes, leading to increases in GBS and NGBS in cabbage lines resistant to blackleg disease

Experimental evidence suggests that *MYB34* genes directly regulate the biosynthesis of indolic glucosinolates in *Arabidopsis thaliana* (Frerigmann and Gigolashvili, 2014) and *Brassica oleracea* (Robin et al., 2016; Yi et al., 2016). *MYB34* together with *MYB51* and *MYB122* provided resistance to the necrotrophic fungal pathogen *Plectosphaerella cucumerina* through the biosynthesis of indolic glucosinolates, where PEN2 plays a key role in activating indolic glucosinolates in response to pathogen attack (Frerigmann et al., 2016). *MYB34* (*Bol007760*) is also induced in broccoli upon methyl jasmonate (MeJA) treatment which is a biotic elicitor that responds to jasmonic acid (JA) signaling (Yi et al., 2016). Likewise in this study, *MYB34* genes *Bol007760* and *Bol017062* showed a 9.35- and 2.13-fold increase, respectively, upon inoculation with 00–100s in the resistant line BN4303 compared to mock-treated plants (Figure 4). Along with the corresponding increase in indolic glucosinolates, this indicates that *MYB34* genes could play a role in the trans-activation of genes necessary for indolic glucosinolate biosynthesis in response to *L. maculans* infection (Figure 2). Consistent with our results, upregulation of *MYB34* (*Bol017062*) in cabbage plants in a previous study was associated with the accumulation of indolic NGBS, which is a derivative of another indolic glucosinolate GBS (Robin et al., 2016).

Some notable *MYB* genes, *MYB34* (*Bol007760*), *MYB28* (*Bol017019, Bol036743*), and *MYB29* (*Bol008849*), as well as other genes such as *ST5b, ST5c* and *FMOGS-OX5* showed higher expression, in general, in BN4098 whereas *CYP81F1* (*Bol017375, BOl017376*) genes showed comparatively higher expression in BN4072. However, these differences in expression did not lead to significant differences in glucosinolate accumulation of the respective indolic and aliphatic glucosinolates, i.e., glucosinolate accumulation was comparable in those lines to the other lines. Hence, this observation is a subject for further investigation. In a recent study, cabbage line BN3383 showed higher expression of *MYB29* (*Bol008849*), *ST5c* and *FMOGS-OX5* compared to other three cabbage inbred lines but that particular line exhibited comparatively lower levels of total glucosinolates (Robin et al., 2016) indicating that expressions of certain biosynthesis genes were not always consistent with higher accumulation of particular glucosinolates from the relevant biosynthesis pathways.

### Accumulation of indolic GBS in cabbage lines resistant to blackleg disease is triggered by increased expression of biosynthesis genes

*ST5a* genes take part in secondary modifications of desulfo-glucosinolates for the biosynthesis of GBS (Figure S1, Liu et al., 2014; Yi et al., 2015). Concentrations of both GBS and NGBS, which have a proven role in antifungal responses in plants, increased in the resistant line BN4303 in response to 00–100s, and this was associated with a remarkable upregulation of *ST5a* (*Bol026200*), *CYP81F4* (*Bol032712*), *CYP81F4* (*Bol032714*), and *CYP81F2* (*Bol026044*) (Figure 5). In a previous study, treatment with MeJA led to a 2,400-fold increase in *CYP81F4* in broccoli and a 10-fold increase in cabbage (Yi et al., 2016), suggesting that resistance is mediated via signaling pathways involved in indolic glucosinolate metabolism. *ST5a* (*Bol026200*) and *CYP81F2* (*Bol026044*) were also upregulated in the moderately resistant interaction between BN4303 and 03-02s and *CYP81F4* (*Bol032712*) was upregulated in the moderately resistant BN4098 × 00–100 s interaction (Figure 5), suggesting that these genes are associated with the enhanced accumulation of indolic glucosinolates in these interactions. Sotelo et al. (2016) also reported a similar association between *CYP81F2* expression and GBS levels in *B. oleracea*.

By contrast, accumulation of indolic GBS and NGBS and few other aliphatic glucosinolates were noted in susceptible combinations, coupled with upregulation of some notable genes, for example: *CYP81F4* genes (Figures 6A,C). These observations raise the question of whether the abundance of intact glucosinolates at a particular time point always reflects their physiological functions. Abundance of glucosinolates in leaf tissues at a particular time-point can be a consequence of simultaneous activation of biosynthesis and catabolism by myrosinases, which can provide upregulation of the bioactive form of the compounds at a particular time point.

### Increased expression of *CYP81F2* leads to accumulation of MGBS and is associated with moderate blackleg resistance in adult cabbage plants

MGBS levels have previously been reported to increase by 30–47% in response to *L. maculans* infection in *Brassica napus* after 5–8 days of inoculation (Wretblad and Dixelius, 2000). In an *in vitro* study, Mithen et al. (1986) also reported anti-fungal activity for MGBS along with two other glucosinolates, SIN and GBS. Here, we observed an increase in MGBS in BN4098 after 00–100s inoculation (Figure 2). The facts that BN4098 showed moderate resistance to 00–100 s and that levels of none of the aliphatic glucosinolates increased suggest a role for MGBS in conferring resistance in this particular interaction. Since *CYP81F2* (*Bol026004*) and *CYP81F4* (*Bol032712, Bol032714*), which are involved in methoxylation and the conversion of GBS to 4-MGBS, are up-regulated in this genotype × isolate combination, it is likely that both the gene and the glucosinolate play roles in conferring resistance to BN4098 to 00–100 s. Our conclusion is consistent with findings of Bednarek et al. (2009) that *CYP81F2* (*Bol026004*) and the myrosinase PEN2 induce antifungal defense (Figure 5).

## Conclusions

Glucosinolate profiling and expression analysis of glucosinolate-related genes in response to blackleg infection identified a direct association between the genes and their corresponding glucosinolates in 3-month-old cabbage plants as supported by both heat map and correlation analyses. This study revealed that the simultaneous, pathogen-induced accumulation of both aliphatic GIV and GER and indolic GBS and NGBS were associated with resistance to blackleg disease in a genotype-specific manner in cabbage. Different glucosinolate profiles were associated with different levels of resistance in a genotype- and isolate-specific manner. Although the presence of certain glucosinolates of either the aliphatic or indolic class was associated with moderate resistance, only the presence of both was associated with complete resistance. The glucosinolates and their corresponding genes identified in this study are candidate genetic and biochemical determinants of resistance and could be tested in efforts to improve blackleg resistance in cabbage.

## Author contributions

IN, JP, and AR conceived of and designed the study. AR managed and inoculated the experimental plants, collected samples, prepared cDNA, performed the qPCR analysis, prepared samples for HPLC and wrote the manuscript. MH carefully commented on a draft of the manuscript. HK conducted the HPLC analysis. GY and RL assisted with the cDNA preparation and qPCR analysis.

### Conflict of interest statement

The authors declare that the research was conducted in the absence of any commercial or financial relationships that could be construed as a potential conflict of interest.
