# Supplementary material for: Leptosphaeria maculans Alters Glucosinolate Profiles in Blackleg Disease–Resistant and -Susceptible Cabbage Lines
Source: Front Plant Sci. 2017 Oct 12;8:1769. doi: 10.3389/fpls.2017.01769 (PMC5644266; doi:10.3389/fpls.2017.01769)
Supplement: Supplementary file 1 [file Table1.DOCX]

# Supplementary Information

**Supplementary Appendix 1.** Brief summary of the analytical pipeline.

**One way ANOVA in Minitab**

Stat — ANOVA — one-way — Response (gene expression / glucosinolate content) and factor (combination)

Posthoc analysis

Comparisons — Tukey — Grouping information — 95% confidence level

**Pearson Correlation analysis in Minitab**

MTB> Corr (variables)

**Heat map analysis in Excel**

Home — Conditional formatting — colour scales

Table S1 Primer sequences for 38 glucosinolate-biosynthesis related genes used for relative expression analysis through qPCR in 3-month-old cabbage plants inoculated with 03-02s and 00-100s isolates (Robin et al. 2016 and Yi et al. 2016).

| **Gene  Name** | **Accession  Number** | **cDNA  Size (bp)** | **Forward Primer Sequence** | **Reverse Primer Sequence** | **Product  Size (bp)** |
| --- | --- | --- | --- | --- | --- |
|  |  |  | Transcription factor-related genes (11 genes) |  |  |
| *MYB28* | Bol007795 | 558 | CCACACCAGTTCAGAGAGGT | GGGAAATGGATCGAAGTCAGC | 221 |
|  | Bol036286 | 615 | GAAGGTAGCTTGAATGCTAATAC | ATTCATGTAGTGCTCCTCATTC | 249 |
|  | Bol017019 | 426 | GTTGCGGCTAAGGTCACTTCT | CAGAAGTAGCGTTGATCTCATGC | 223 |
|  | Bol036743 | 426 | CTTGGGCGCTGCTACATTAC | ATCGTTCTCCTCGTTGTGGT | 241 |
| *MYB29* | Bol008849 | 513 | CGCCCAAGACTTCTGAGTT | TGATATTGCCCATGGAAGCTG | 234 |
| *MYB34* | Bol007760 | 843 | TG‍AAGGAGGATGGCGTACTC | CAGTTCGTCCCGCCAAATTA | 203 |
|  | Bol017062 | 951 | AAGGTGGATGGCGTACTCTC | TGTGAGTGGTTGGATCGACA | 279 |
|  | Bol036262 | 294 | CCCCGAGTTCTTTAGCAACC | TCCAAGTCCAGATCGTCTTCT | 198 |
| *MYB51* | Bol013207 | 1002 | CCAGAGATTCCAGAGAAGC | CAAGTCACACTGCTACTACTAC | 233 |
|  | Bol030761 | 990 | CAGACAACTATATCGAGTAACG | TATCATTAACGGTCATCTGG | 274 |
| *MYB122* | Bol026204 | 981 | GACCATTCCGAGACATTGCC | GCATCGTGGATCATGTGGAG | 284 |
|  |  |  | Aliphatic biosynthesis-related genes (10 genes) |  |  |
| *ST5b* | Bol026201 | 1035 | CCGAGCCGTCAGAATTCAAG | GCTATGGCGAAAGTGAGAGC | 247 |
|  | Bol026202 | 1035 | AAGCCTTGACTTTCGCCATC | ACTTCACAACTGAGTCCGGT | 204 |
| *ST5c* | Bol030757 | 1014 | CCACGCCCAAAACTTCTTCA | TGAGTGGAGAAGAGCGTGTT | 246 |
| *FMOGS-OX2* | Bol010993 | 1386 | GAGAAGGTATCCGAGCCACA | GTCCACTGCAAACAACGACT | 200 |
| *FMOGS-OX5* | Bol029100 | 1347 | CTTGCTCCAACGCTTTCCTT | CCTCAGCTCTCCAGTGTTCA | 280 |
|  | Bol031350 | 1380 | GACACTACACAGAGCCTCGT | CCCCGGGAAGCTTCTCATAT | 234 |
| *AOP2* | Bo2g102190 |  | GGAACGTGTCTCCAAAACCC | TAGCACCATCACCAGCATCA | 354 |
|  | Bo3g052110 |  | CCAGGAAGTGAGAAGTGGGT | ACCAACATCCGCACCAGTAT | 552 |
|  | Bo9g006240 |  | CCAGGAAGTGAGAAGTGGGT | TAGCACCATCACCAGCATCA | 517 |
| *GSL-OH* | Bol033373 | 243 | GATTGTGCAAAAGGCTTGT | AGAGCATTAGGATTAGGAGGA | 188 |
|  |  |  | Indolic biosynthesis-related genes (17 genes) |  |  |
| *ST5a* | Bol026200 | 1017 | GTCCGGTTGCAAGATGGTTT | CCTCTCCGGGTTCTCTTTGT | 214 |
|  | Bol039395 | 1014 | TGCCGTTTGTGAAGAGGTTG | CCCAATCTCCAACCTTCCCT | 210 |
| *CYP81F4* | Bol032712 | 1506 | CGGTGGAGGAGAAGGAGAAA | CTGACACATGGCTCGTAACG | 226 |
|  | Bol032714 | 960 | ACCCTGGTGAATACTTGCCA | GAAACACACTGAAGCAGAAC | 239 |
|  | Bol028918 | 1503 | GTTTGCGGCATCAGAGACAT | GAATAGTCCACGCGTTCACC | 299 |
| *CYP81F1* | Bol017375 | 369 | AAGCAGAGCGGTTCAAGAAG | GCGTGACCATTGTGTTACCA | 204 |
|  | Bol017376 | 246 | CCGTCTCCTTCAACGGTTCT | CGACGTATTTACCGGTGAGC | 170 |
|  | Bol028913 | 1500 | GAGACCTCCGCAGTAACCTT | GTCCTCCGTCGGTCTTCTAG | 222 |
|  | Bol028914 | 1497 | CTTTCCAACTGACGGCCAAA | CGTTAGGTCCGAGAAAAGCG | 257 |
| *CYP81F2* | Bol012237 | 933 | GCAGCCGTGACACTAGAATG | TCCGCCAATCTTGAGGTCTT | 231 |
|  | Bol014239 | 1482 | TTGTACCGCGTTCTCCTTCT | GACACCATCCTCTGACCCAA | 238 |
|  | Bol026044 | 1482 | TTCTCCCTACGTTACGGCTC | CTACGAACGGAGAGGAGTCC | 251 |
| *CYP81F3* | Bol028919 | 1500 | TAACAGCGGAGGAGAAGACG | CACCTTCTAACTGGGCCTGA | 260 |
|  | Bol032711 | 1492 | CCGTCTCACCAACTTCCTCT | CTTCTCAAAGCTCCCTCCCA | 292 |
| *IGMT1* | Bol007029 | 1119 | GTGTTCCTCTCACCTTCCGA | GTGTTGAGGAAGACGCTGTC | 260 |
|  | Bol020663 | 342 | AGATGCCATGATCTTGAAACGT | CCAGCAATGATAAGCCTGACA | 298 |
| *IGMT2* | Bol007030 | 1125 | AGCCTTTCCCATGGTTCTCA | TCTCTCGCCCTTTCCAAACT | 223 |

Table S2 Degrees of freedom, test statistic and p-values for glucosinolate content and expression of glucosinolate biosynthesis genes in blackleg disease-resistant and -susceptible 3-month-old cabbage plants.

| **Variable** | | **df** | **F value** | **P value** |
| --- | --- | --- | --- | --- |
| **Glucosinolate contents** | |  |  |  |
| Progoitrin | | 15 | 64.7 | <0.01 |
| Glucoiberin | | 15 | 33.99 | <0.01 |
| Sinigrin | | 15 | 305.3 | <0.01 |
| Gluconapin | | 15 | 276.3 | <0.01 |
| Glucoiberverin | | 15 | 43.8 | <0.01 |
| Glucoerucin | | 15 | 141.0 | <0.01 |
| Glucobrassicin | | 15 | 31.04 | <0.01 |
| Neo-glucobrassicin | | 15 | 79.38 | <0.01 |
| Methoxy-glucobrassicin | | 15 | 27.28 | <0.01 |
| 4-Hydroxy-glucobrassicin | | 15 | 2334 | <0.01 |
| Total glucosinolate | | 15 | 104.14 | <0.01 |
| **Relative expression of genes** | |  |  |  |
| *MYB28* | Bol007795 | 15 | 68.6 | <0.01 |
|  | Bol036286 | 15 | 107.7 | <0.01 |
|  | Bol017019 | 15 | 117.2 | <0.01 |
|  | Bol036743 | 15 | 179.5 | <0.01 |
| *MYB29* | Bol008849 | 15 | 144.1 | <0.01 |
| *MYB34* | Bol007760 | 15 | 297.8 | <0.01 |
|  | Bol017062 | 15 | 199.8 | <0.01 |
|  | Bol036262 | 15 | 200.5 | <0.01 |
| *MYB51* | Bol013207 | 15 | 53.3 | <0.01 |
|  | Bol030761 | 15 | 91.2 | <0.01 |
| *MYB122* | Bol026204 | 15 | 76.8 | <0.01 |
| *ST5b* | Bol026201 | 15 | 140.5 | <0.01 |
|  | Bol026202 | 15 | 62.8 | <0.01 |
| *ST5c* | Bol030757 | 15 | 56.5 | <0.01 |
| *FMOGS-OX2* | Bol010993 | 15 | 68.4 | <0.01 |
| *FMOGS-OX5* | Bol029100 | 15 | 26.8 | <0.01 |
|  | Bol031350 | 15 | 38.1 | <0.01 |
| *AOP2* | Bo2g102190 | 15 | 68.2 | <0.01 |
|  | Bo3g052110 | 15 | 108 | <0.01 |
|  | Bo9g006240 | 15 | 44.5 | <0.01 |
| *GSL-OH* | Bol033373 | 15 | 47.1 | <0.01 |
| *ST5a* | Bol026200 | 15 | 69.6 | <0.01 |
|  | Bol039395 | 15 | 304.3 | <0.01 |
| *CYP81F4* | Bol032712 | 15 | 48.7 | <0.01 |
|  | Bol032714 | 15 | 98.2 | <0.01 |
|  | Bol028918 | 15 | 21.3 | <0.01 |
| *CYP81F1* | Bol017375 | 15 | 127.6 | <0.01 |
|  | Bol017376 | 15 | 76.2 | <0.01 |
|  | Bol028913 | 15 | 36.5 | <0.01 |
|  | Bol028914 | 15 | 81.9 | <0.01 |
| *CYP81F2* | Bol012237 | 15 | 39.8 | <0.01 |
|  | Bol014239 | 15 | 59.9 | <0.01 |
|  | Bol026044 | 15 | 150.6 | <0.01 |
| *CYP81F3* | Bol028919 | 15 | 31.5 | <0.01 |
|  | Bol032711 | 15 | 222.6 | <0.01 |
| *IGMT1* | Bol007029 | 15 | 69.7 | <0.01 |
|  | Bol020663 | 15 | 58.1 | <0.01 |
| *IGMT2* | Bol007030 | 15 | 941.2 | <0.01 |

**
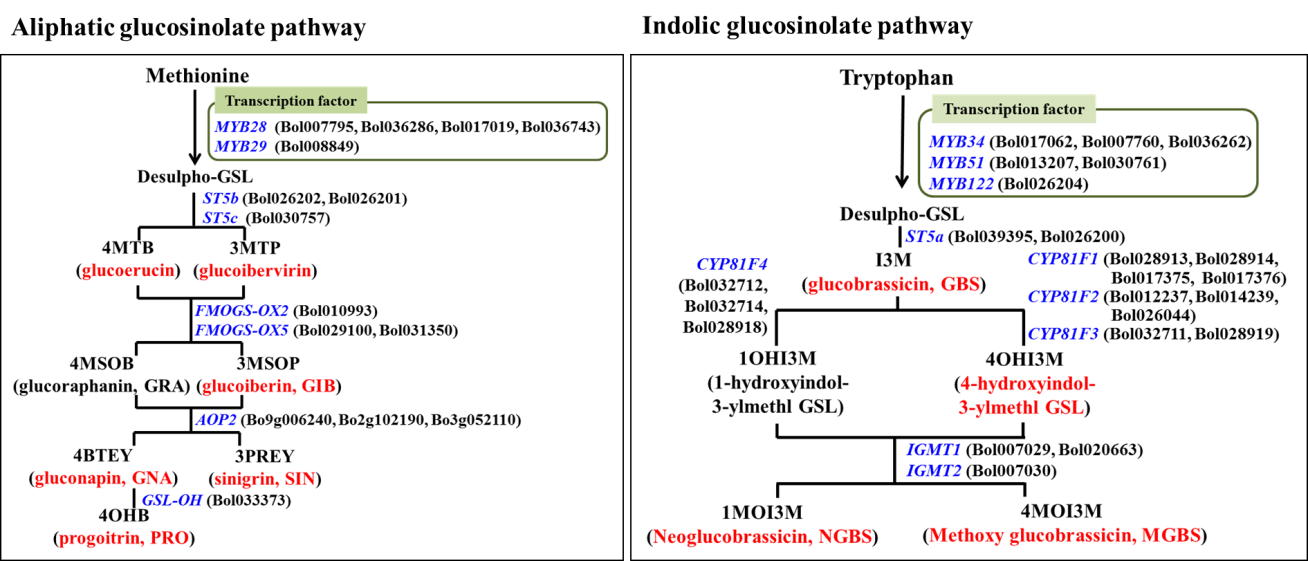
**

Figure S1 The glucosinolate biosynthesis and related transcription factor genes (blue letters) analyzed in this work, with their positions in the aliphatic and indolic glucosinolate (GSL) biosynthesis pathways indicated (Adopted from Yi et al. 2016). A total of 15 and 23 genes were selected from the aliphatic and indolic glucosinolate biosynthesis pathways, respectively. Glucosinolate compounds in red letters were identified through HPLC in this study.

**
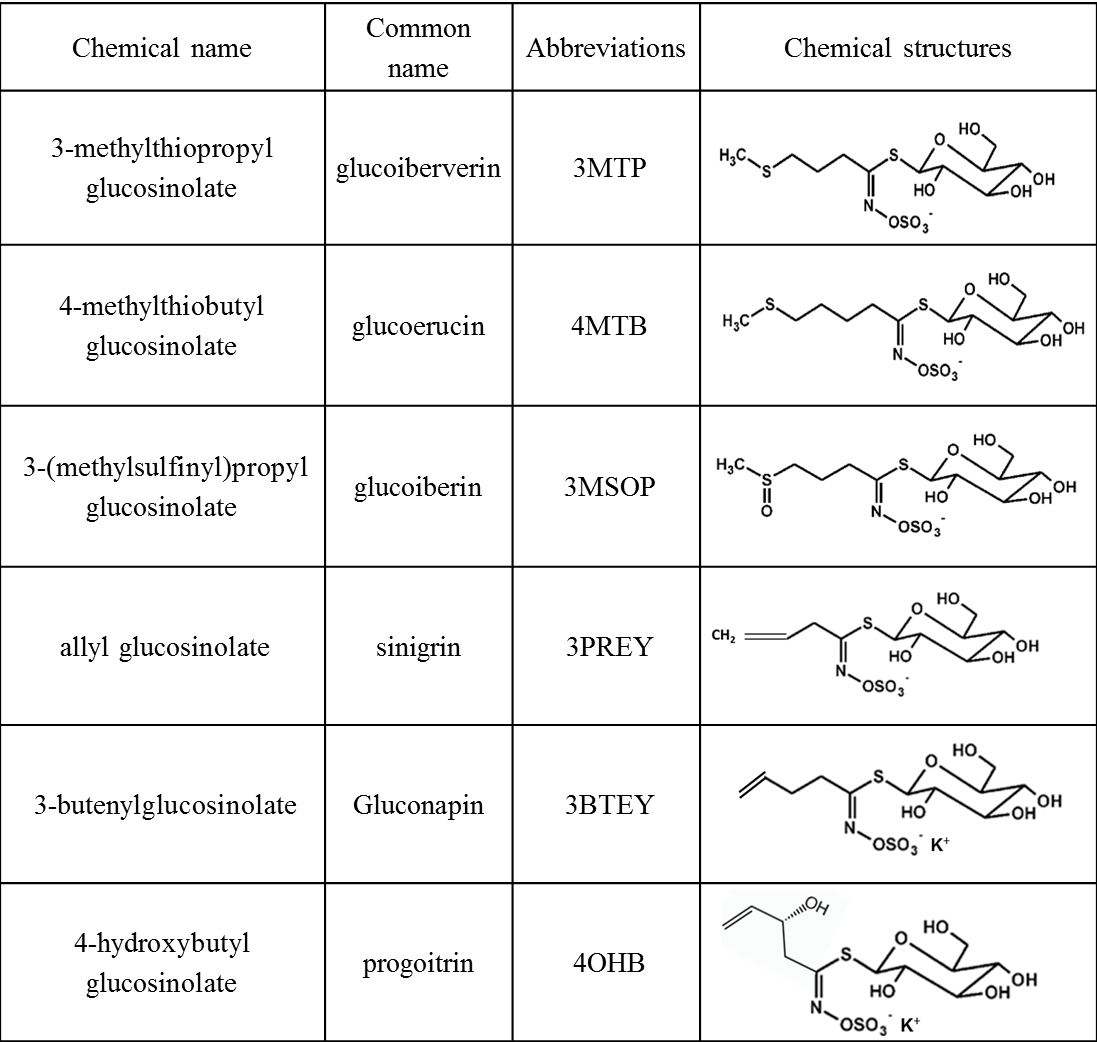
**

**(a)**

**
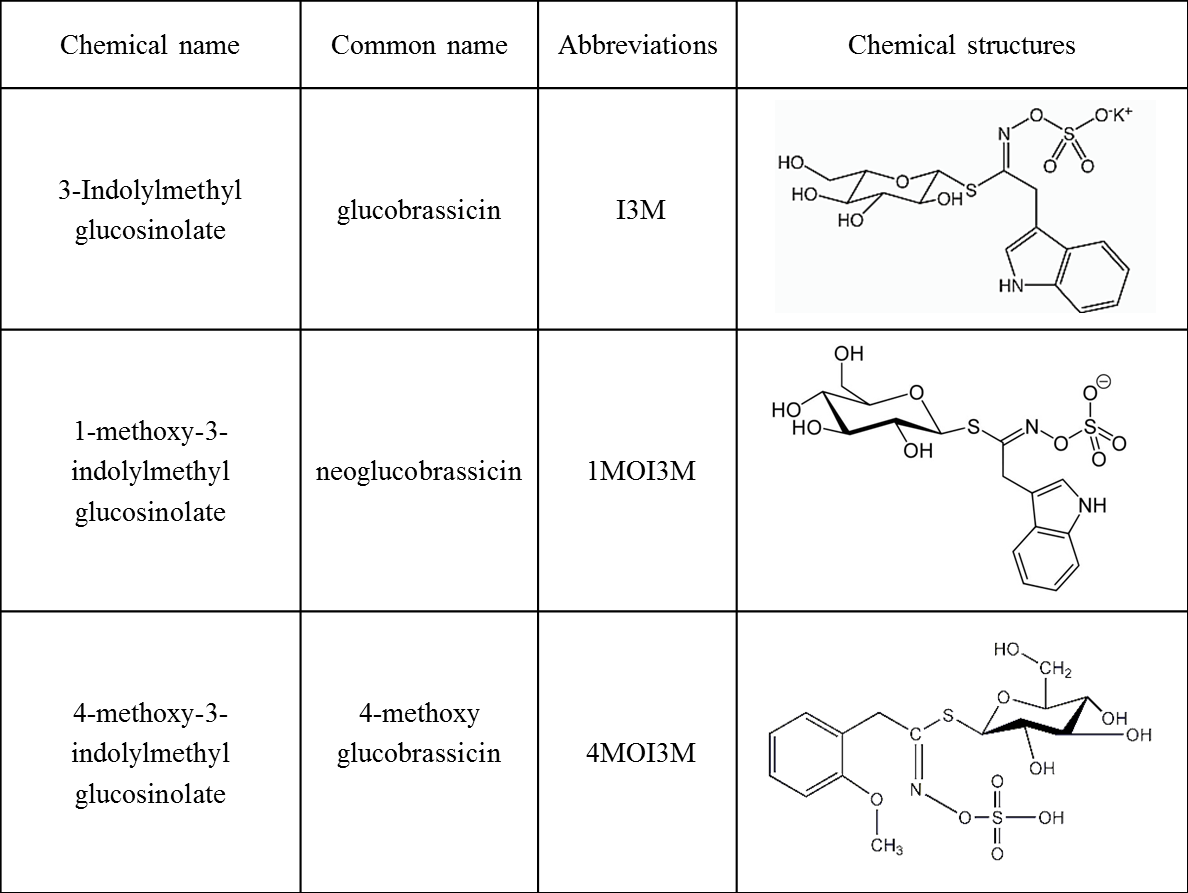
**

**(b)**

**Figure S2.** Chemical structures of the glucosinolate compounds and intermediates. a) aliphatic glucosinolates, b) indolic glucosinolates.

**
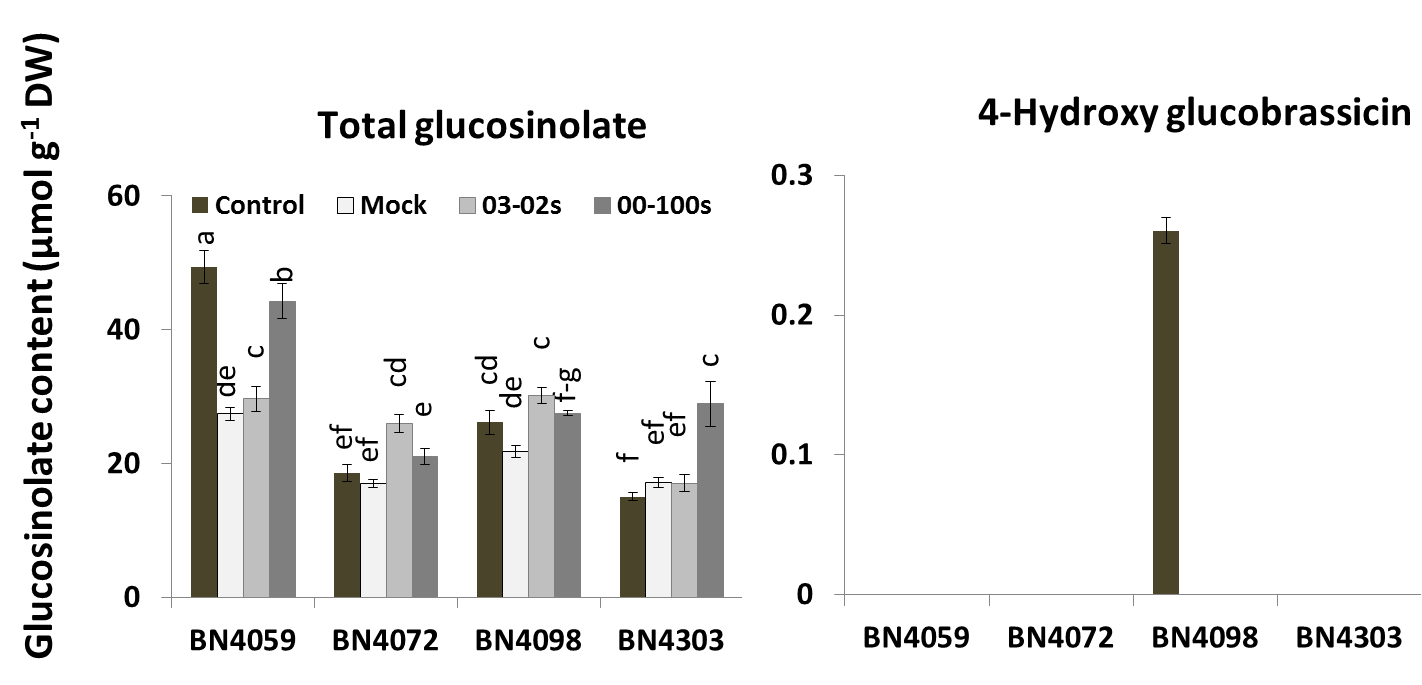
**

Figure S3 Total glucosinolate and 4-hydroxyglucobrassicin contents in leaf samples from the four cabbage lines (BN4059, BN4072, BN4098, and BN4303) under four different treatments (control, mock, inoculation with 03-02s or 00-100s) four days after inoculation. The mean of three biological replicates is presented. Vertical bars indicate standard error. Different letters indicate statistically significant differences between genotype × treatment combinations.

**
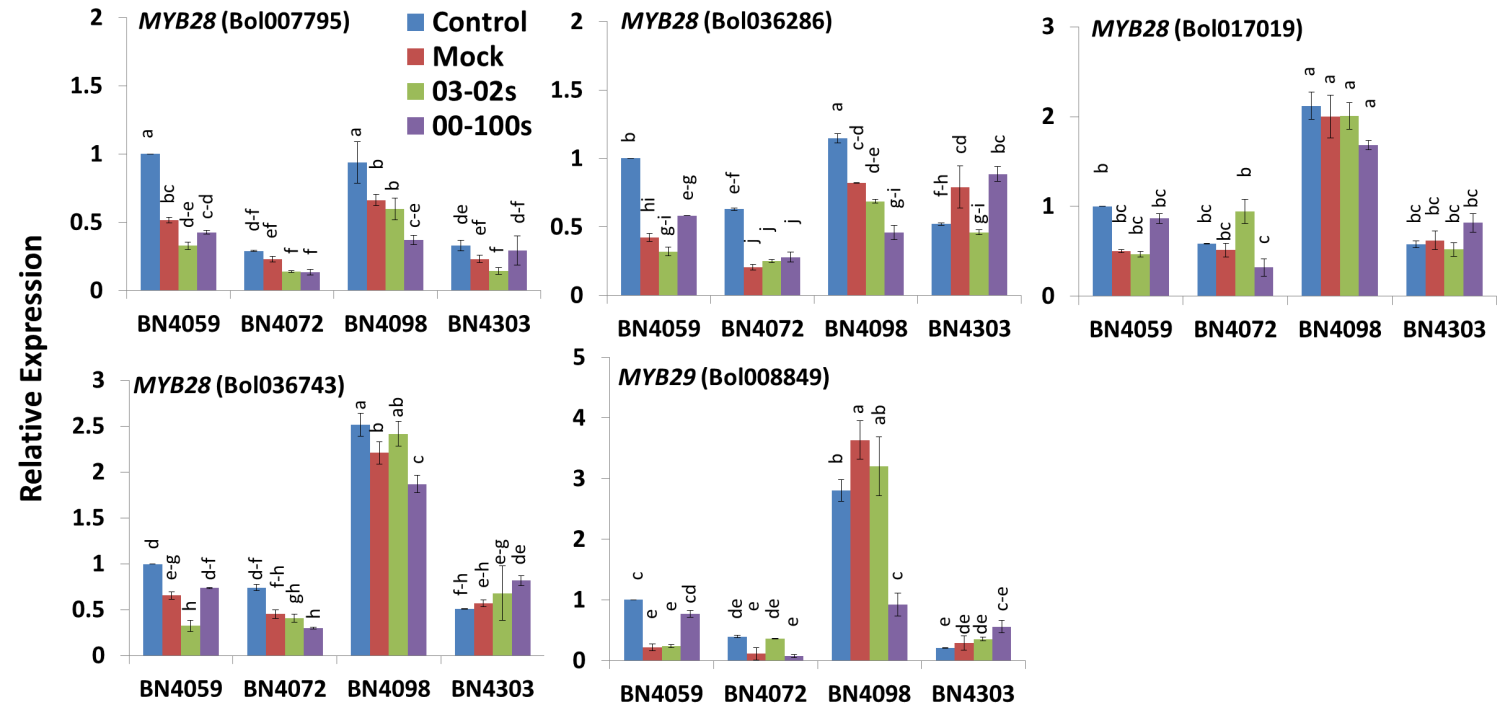
**

Figure S4 Relative expression of aliphatic glucosinolate transcription factor-related genes in leaf samples from the four cabbage lines (BN4059, BN4072, BN4098, and BN4303) under four different treatments (control, mock, inoculation with 03-02s or 00-100s) four days after inoculation. The mean of three biological replicates is presented. Vertical bars indicate standard error. Different letters indicate statistically significant differences between genotype × treatment combinations.

**
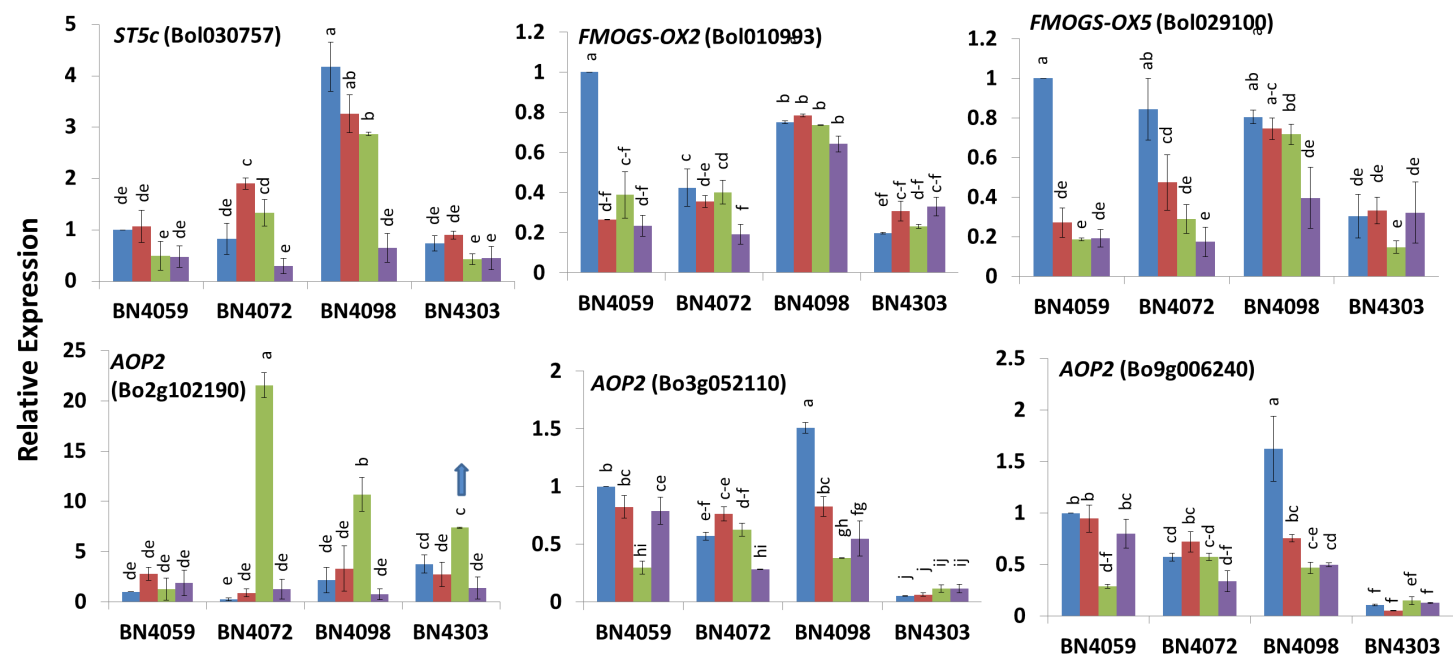
**

Figure S5 Relative expression of aliphatic glucosinolate biosynthesis genes in leaf samples from the four cabbage lines (BN4059, BN4072, BN4098, and BN4303) under four different treatments (control, mock, inoculation with 03-02s or 00-100s) four days after inoculation. The mean of three biological replicates is presented. Vertical bars indicate standard error. Different letters indicate statistically significant differences between genotype × treatment combinations.

**
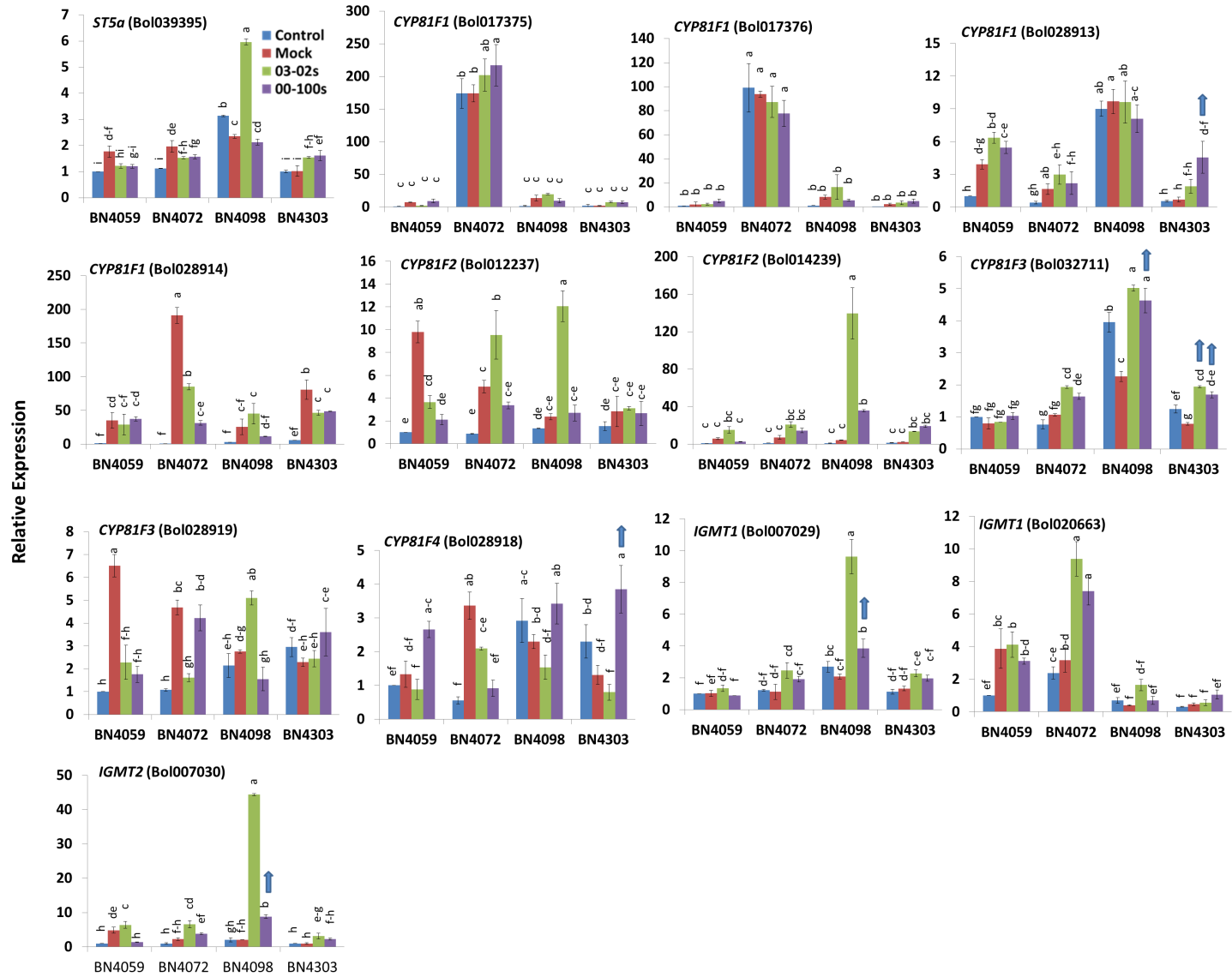
**

Figure S6 Relative expression of indolic glucosinolate biosynthesis genes in leaf samples from the four cabbage lines (BN4059, BN4072, BN4098, and BN4303) under four different treatments (control, mock, inoculation with 03-02s or 00-100s) four days after inoculation. The mean of three biological replicates is presented. Vertical bars indicate standard error. Different letters indicate statistically significant differences between genotype × treatment combinations.
